# BRCA1 and TP53 codeficiency causes a PARP inhibitor–sensitive erythroproliferative neoplasm

**DOI:** 10.1172/jci.insight.158257

**Published:** 2022-12-22

**Authors:** Gerardo Lopez-Perez, Ranjula Wijayatunge, Kelly B. McCrum, Sam R. Holmstrom, Victoria E. Mgbemena, Theodora S. Ross

**Affiliations:** Department of Internal Medicine, University of Texas (UT) Southwestern Medical Center, Dallas, Texas, USA.

**Keywords:** Hematology, Leukemias, Tumor suppressors, p53

## Abstract

Mutations in the *BRCA1* tumor suppressor gene, such as 5382insC (*BRCA1*^insC^), give carriers an increased risk for breast, ovarian, prostate, and pancreatic cancers. We have previously reported that, in mice, *Brca1* deficiency in the hematopoietic system leads to pancytopenia and, as a result, early lethality. We explored the cellular consequences of *Brca1*-null and *BRCA1*^insC^ alleles in combination with *Trp53* deficiency in the murine hematopoietic system. We found that *Brca1* and *Trp53* codeficiency led to a highly penetrant erythroproliferative disorder that is characterized by hepatosplenomegaly and by expanded megakaryocyte erythroid progenitor (MEP) and immature erythroid blast populations. The expanded erythroid progenitor populations in both BM and spleen had the capacity to transmit the disease into secondary mouse recipients, suggesting that *Brca1* and *Trp53* codeficiency provides a murine model of hematopoietic neoplasia. This *Brca1*/*Trp53* model replicated Poly (ADP-ribose) polymerase (PARP) inhibitor olaparib sensitivity seen in existing *Brca1*/*Trp53* breast cancer models and had the benefits of monitoring disease progression and drug responses via peripheral blood analyses without sacrificing experimental animals. In addition, this erythroid neoplasia developed much faster than murine breast cancer, allowing for increased efficiency of future preclinical studies.

## Introduction

*BRCA1* is a tumor suppressor gene that encodes for a DNA repair protein involved in double-strand break (DSB) repair. Inherited mutations in *BRCA1* are predisposed to an autosomal dominant predisposition to breast, ovarian, and other cancers. In the absence of BRCA1, cells are unable to accurately repair DNA DSBs by homologous recombination (HR) (1–3) leading to genomic instability and cancer predisposition. *BRCA1* mutant cancer cells rely on alternative DNA-repair pathways in the absence of HR. Poly(ADP-ribose) polymerase (PARP) is an enzyme involved the single-strand break (SSB) repair pathway (4) that *BRCA1* mutant cells may rely on. This dependency leads to a synthetic lethal interaction between BRCA1 deficiency and PARP inhibition, where increased genomic instability leads to cell death (5–7). PARP inhibitors are used to treat *BRCA1*-mutated ovarian, prostate, and pancreatic cancers (8).

Knowledge of the role of BRCA1 in the hematopoietic system is lacking, despite human data associating BRCA1 to hematologic malignancies. For example, *BRCA1* mutations have been linked to Fanconi anemia (FA), an inherited BM failure syndrome with hematopoietic phenotypes such as myelodysplastic syndrome, cytopenia, and acute myeloid leukemia (AML), since BRCA1 interacts with several FA proteins (9–13). *BRCA1* mutations are detected in AML patients (although at a low frequency) (14), and BRCA1 expression is decreased in chronic myelogenous leukemia (CML) cells (15, 16). There are studies that suggest patients carrying *BRCA1* mutations have shown more adverse hematologic outcomes following chemotherapy compared with *BRCA2* mutation carriers or noncarriers (17, 18). These data suggest that BRCA1 is an important regulator of hematopoiesis in humans.

Prior publications by us and others identified that the conditional deletion of *Brca1* leads to hematopoietic defects in mice (17, 19). We established a requirement for Brca1 in the murine hematopoietic system where hematopoietic stem cell (HSC) and progenitor cell–specific deletion of *Brca1* under the control of either *Vav1*-*iCre* or *Mx1*-*Cre* transgenes led to BM failure and early lethality (17). This *Brca1* null phenotype was worsened by compound heterozygosity of the null allele with the humanized *BRCA1* 5382insC (*BRCA1*^insC^) allele. The *BRCA1*^insC^ mutation is one of the relatively common Ashkenazi Jewish *BRCA1* founder mutations that are known to increase the risk for cancer (20). This allele is a knockin of human *BRCA1* cDNA carrying the 5382insC mutation into the mouse *Brca1* locus. Thus, the *BRCA1*^insC^ allele is constitutively expressed under the control of endogenous mouse regulatory elements. Our data demonstrate that the *BRCA1*^insC^ allele is more detrimental to hematopoietic tissue than a simple *Brca1* null allele.

In further exploration to the roles of Brca1 in hematopoeisis, we have now made the discovery that hematopoietic *Brca1* deficiency in combination with *Trp53* deficiency leads to an erythroid neoplasia. In mice, *Trp53* germline nullizygosity predominantly leads to T cell lymphomas at 3–5 months of age, and heterozygous mice develop a greater spectrum of cancers (including lymphomas and sarcomas) but at a much later age than the homozygotes (21–23). However, *Trp53* mutations have been shown to induce nonlymphoid neoplasms in combination with a variety of genetic lesions (22, 24–27). None have directly evaluated the outcomes of combined *Brca1* and *Trp53* deficiency in the hematopoietic system.

In this report, we characterize a *Brca1*/*Trp53* double deficiency–associated hematopoietic neoplasia, using *Brca1*-null and *BRCA1*^insC^ mutant alleles. As was observed with Brca1 single deficiency, *Brca1/Trp53* double deficiency was more detrimental to mice carrying the 5382insC allele compared with mice with only the null allele. *Brca1/Trp53* double deficiency–associated erythroid neoplasia is a rapid onset, highly penetrant condition and can be transplanted to multiple syngenic immune-replete mice. As expected from a *Brca1* deficiency–associated neoplasia, PARP inhibitor olaparib treatment attenuated disease phenotypes. This mouse model could allow for rapid in vivo screening for compounds of therapeutic potential — and even for the assessment of pathogenicity of *BRCA1* variants of uncertain significance (VUS).

## Results

### Brca1 and Trp53 double deficiency in the BM leads to an erythroproliferative disorder.

**Figure 1:**
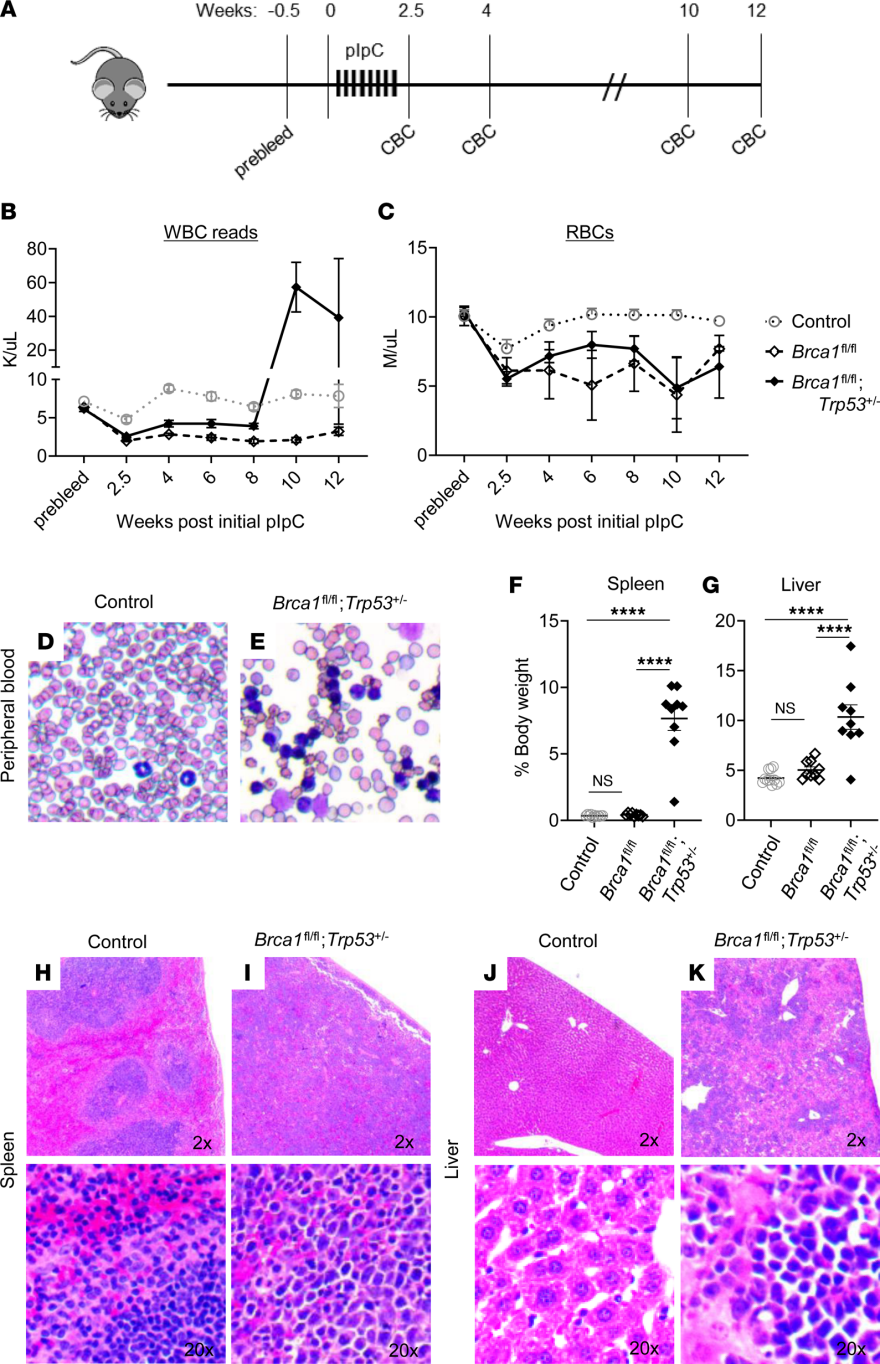
*Brca1/Trp53*–double deficient *Mx1-Cre;Brca1*^fl/fl^;*Trp53*^+/–^ mice develop a rapid and robust hematological disease. (**A**) Schematic of experimental design. Control, *Mx1-Cre;Brca1*^fl/fl^ and *Mx1-Cre;Brca1*^fl/fl^;*Trp53*^+/–^ were treated with pIpC and monitored for up to 12 weeks after initial pIpC treatment. (**B** and **C**) Average peripheral blood WBC reads (indicative of nonleukocyte, nucleated cells) (**B**) and RBC (**C**) counts before (prebleed) and after initial pIpC treatment. Control (*n* = 13), *Mx1-Cre;Brca1*^fl/fl^ (*n* = 11), and *Mx1-Cre;Brca1*^fl/fl^;*Trp53*^+/–^ (*n* = 9). (**D** and **E**) Wright-Giemsa–stained peripheral blood smears from age-matched control and *Mx1-Cre;Brca1*^fl/fl^;*Trp53*^+/–^ mice. (**F** and **G**) Spleen and liver weights at moribund/terminal stage or 12 weeks after initial pIpC treatment. Control (*n* = 11), *Mx1-Cre;Brca1*^fl/fl^ (*n* = 9), and *Mx1-Cre;Brca1*^fl/fl^;*Trp53*^+/–^ (*n* = 9). (**H**–**K**) Representative H&E-stained sections of effaced spleens (**I**) and infiltrated livers (**K**) of *Mx1-Cre;Brca1*^fl/fl^; *Trp53*^+/–^ mice compared with control (**H** and **J**) mice. Data represent mean ± SEM. Statistical significance was assessed using 1-way ANOVA followed by Bonferroni correction (****P < 0.00003). Controls were without *Mx1-Cre*; all other mice carry *Mx1-Cre*.

In this study, we investigated the compound effects of *Brca1* and *Trp53* deficiencies in hematopoietic tissue. We used the *Mx1-Cre* system (28), where induction of interferon following injection with polyinosinic:polycytidylic acid (pIpC) drives Cre-mediated recombination in hematopoietic stem and progenitor cells to generate null alleles of *Brca1* in hematopoietic tissues (Figure 1A). To confirm deletion of the *Brca1* floxed allele and the generation of the null allele following pIpC treatment, we tested for loss of the nonrecombined, floxed allele of *Brca1* (Supplemental Figure 1; supplemental material available online with this article; https://doi.org/10.1172/jci.insight.158257DS1). The amount of this allele in *Mx1-Cre;Brca1*^fl/fl^;*Trp53*^+/–^ spleens was significantly decreased compared with control *Brca1*^fl/fl^;*Trp53*^+/–^ spleens (*P* = 0.003), confirming recombination of the conditional *Brca1* allele. As predicted, no recombination was seen in *Mx1-Cre;Brca1*^fl/fl^;*Trp53*^+/–^ and control *Brca1*^fl/fl^;*Trp53*^+/–^ brain tissue. These data confirm the hematopoietic tissue–specific deletion of *Brca1* following pIpC treatment and, thus, the generation of a hematopoietic tissue–specific *Brca1/Trp53* double deficiency model.

Using this model, we compared the hematopoietic phenotypes of double *Brca1/Trp53* deficiency with those of single Brca1 deficiency. As observed in prior work (17), *Brca1* deficiency in a *Trp53* WT background led to early mortality and pancytopenia, including low WBC and RBC counts. No significant difference in survival was seen between *Mx1*-*Cre*;*Brca1*^fl/fl^ and *Mx1*-*Cre*;*Brca1*^fl/fl^;*Trp53*^+/–^ (median survival 11.3 versus 10.6 weeks, respectively) (data not shown). Similarly, *Trp53* heterozygosity did not alter the anemia observed in *Brca1*-null mice (Figure 1C). However, following an initial phase of low WBCs, a majority (77.8%) of *Brca1/Trp53*–double deficient mice developed elevated WBCs (at > 8 weeks after initial pIpC) (Figure 1B and Supplemental Figure 2A). Despite elevated WBC parameters via automated analysis, smears of peripheral blood from the same mice showed that the abundant nucleated cells were not leukocytes but had, in fact, an erythroblast morphology (29) (Figure 1, D and E). Indeed, abnormal RBCs and nucleated RBCs are noted to read as WBCs in automated analyses, according to the manufacture’s manual (30). All *Mx1*-*Cre*;*Brca1*^fl/fl^;*Trp53*^+/–^ mice developed hepatosplenomegaly (Figure 1, F and G). At terminal stage, the spleens of *Mx1-Cre;Brca1*^fl/fl^*;Trp53^+/–^* mice were ~22-fold larger than controls (7.7% versus 0.35% spleen/body weight; 1.78 g versus 0.09 g absolute weight) and the livers ~2-fold larger (10.35% versus 5.0% liver/body weight; 2.4 g versus 1.1 g absolute weight) than controls. Enlarged spleens were effaced without the normal red/white pulp structure (Figure 1, H and I). Similar to spleens, livers of diseased mice showed mononuclear cell infiltration (Figure 1, J and K).

**Figure 2:**
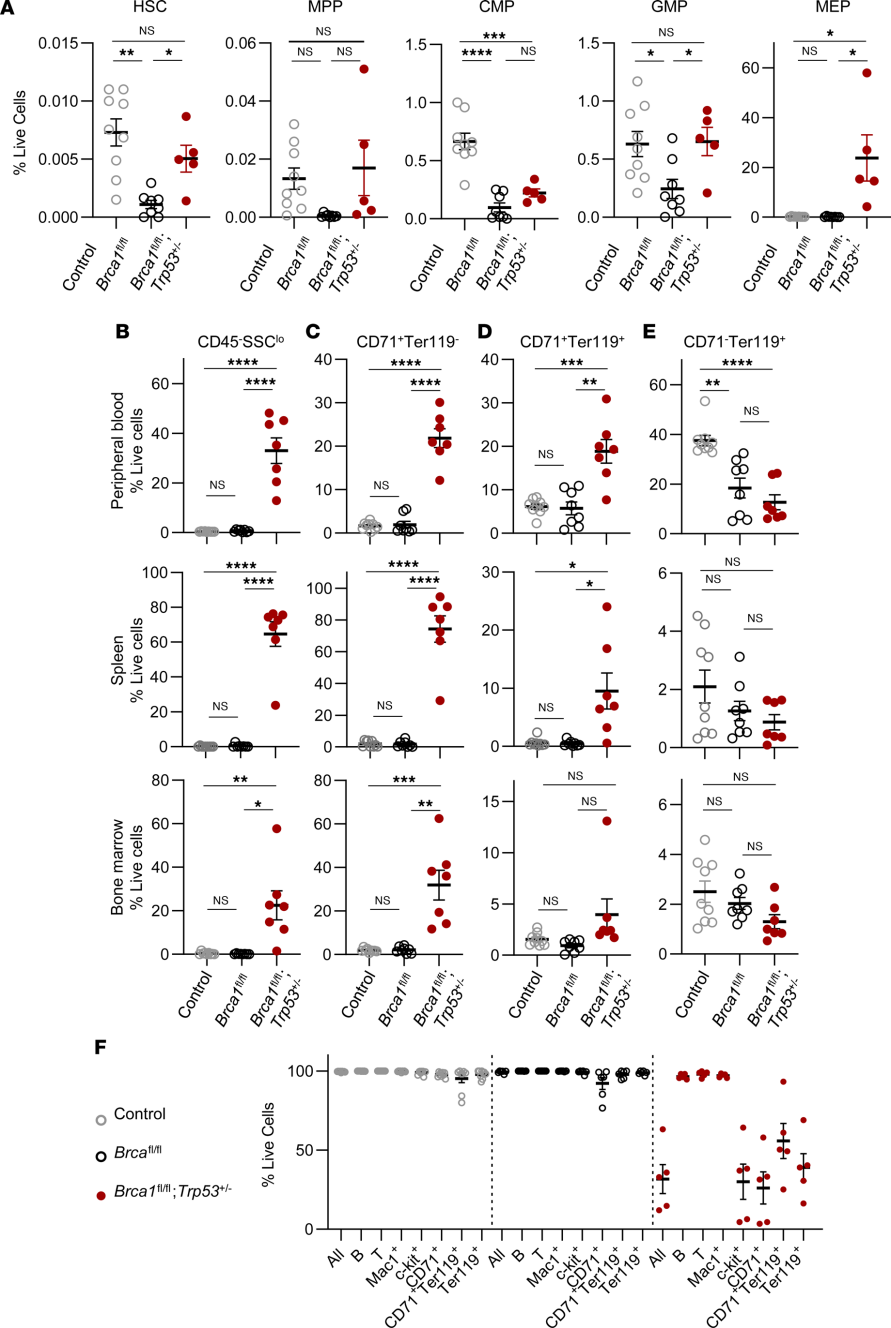
Increased immature erythroblasts in hematopoietic tissue of diseased *Mx1-Cre;Brca1*^fl/fl^;*Trp53*^+/–^ mice. (**A**) Hematopoietic stem cell (HSC), multipotent progenitor (MPP), common myeloid progenitor (CMP), granulocyte monocyte progenitor (GMP), and megakaryocyte/erythrocyte progenitor (MEP) frequencies in the BM of control (*n* = 9), *Mx1-Cre;Brca1*^fl/fl^ (*n* = 8), and *Mx1-Cre;Brca1*^fl/fl^;*Trp53*^+/–^ (*n* = 5) mice. (**B**–**E**) Flow cytometric analysis of peripheral blood (top), spleen (middle), and BM (bottom) for CD45^–^SSC^lo^ erythroblasts (**B**), early CD71^+^Ter119^–^ (**C**), mid CD71^+^Ter119^+^ (**D**), and late CD71^–^Ter119^+^ (**E**) erythroid progenitors in control (*n* = 9), *Mx1-Cre;Brca1*^fl/fl^ (*n* = 8), and *Mx1-Cre;Brca1*^fl/fl^; *Trp53*^+/–^ (*n* = 7) mice. (**F**) Percentage of CD45^+^ cells in spleen B cells, T cells, granulocytes (Mac1^+^), c-kit^+^ cells, and erythroid progenitors (early CD71^+^Ter119^–^, mid CD71^+^Ter119^+^, and late CD71^–^Ter119^+^) in control (*n* = 9), *Mx1-Cre;Brca1*^fl/fl^ (*n* = 8), and *Mx1-Cre;Brca1*^fl/fl^;*Trp53*^+/–^ (*n* = 5) mice. Data represent mean ± SEM. Statistical significance was assessed using 1-way ANOVA followed by Bonferroni correction (*P < 0.0167, **P < 0.003, ***P < 0.0003,****P < 0.00003). Controls were without *Mx1-Cre*; all other mice carry *Mx1-Cre*.

To characterize the cell types contributing to the disease, we analyzed hematopoietic cell compartments by flow cytometry. As we had reported before (17), Brca1 deficiency alone (in *Mx1*-*Cre*;*Brca1*^fl/fl^ mice) led to significant decreases in BM HSC populations (Lineage^–^Sca^+^c-kit^+^ [LSK], CD150^+^CD48^–^), multipotent progenitor populations (MPP; CD150^–^CD48^–^LSK), common myeloid progenitor populations (CMP; Sca^–^LK CD34^+^CD16/32^lo^), and granulocyte/monocyte myeloid progenitor populations (GMP; Sca^–^LK CD34^+^CD16/32^hi^) (Figure 2A). *Trp53* deficiency contributed to modest increases in HSC and progenitor (MPP, CMP, and GMP) frequencies, as would be expected from *Trp53* deficiency alone, through its regulation of cell cycle and apoptosis (26, 31). However, none of these frequencies increased above that of control. On the other hand, the megakaryocyte/erythroid progenitor (MEP; Sca^–^LK^+^CD34^−^CD16/32^lo^) compartment was massively expanded (~110-fold increase, *P* = 0.004) in *Mx1*-*Cre*;*Brca1*^fl/fl^;*Trp53*^+/–^ mice. Consistent with BM cytometry, there was no elevation in B220^+^ B cells, CD3^+^ T cells, or CD11b^+^Gr1^+^ myeloid cells in peripheral blood (Supplemental Figure 2, B–D). Further analysis of peripheral blood, spleen, and BM from diseased *Mx1*-*Cre*;*Brca1*^fl/fl^;*Trp53*^+/–^ mice showed increases in SSC^lo^CD45^–^ gated cells (Figure 2B), which previous studies have shown to correspond to a population that lacks myeloid- or lymphoid-associated surface antigens and that represents nucleated erythroid cells based on high expression of surface transferrin receptor (CD71) and glycoprotein A (Ter119) (32). Indeed the CD71^+^ erythroid cells were elevated in all hematopoietic compartments tested. We further analyzed this expanded erythroid cell population using Ter119 — a marker for late stages of erythropoiesis (33) — and activated cellular stem cell factor receptor c-kit — a marker for early hematopoietic developmental stages (34). A majority of CD71 cells (~80% in BM and spleen) also expressed the c-kit marker, suggesting the immature nature of the erythroid cells (Supplemental Figure 2E). Consistently, early-erythroid (CD71^+^Ter119^–^) and miderythroid (CD71^+^Ter119^+^) progenitor cell frequencies in *Mx1*-*Cre*;*Brca1*^fl/fl^;*Trp53*^+/–^ mice were elevated compared with control and *Mx1*-*Cre*;*Brca1*^fl/fl^ mice (Figure 2, C and D). Despite immature erythroid cell hyperplasia, no corresponding increases were seen in CD71^–^Ter119^+^ late erythroid stage (Figure 2E). Due to the block in differentiation to mature RBCs, a positive feedback cycle of inefficient erythropoiesis and anemia persists. In contrast, *Mx1*-*Cre*;*Brca1*^fl/fl^ mice showed no significant differences from control in frequencies for any of the populations tested. Of the tissues tested, the spleen had the highest frequency of erythroid blast cells, suggesting the spleen as the major site of housing for the hyperplasia. Together, these data suggest that this disease is characterized by a dysregulated erythroid, rather than lymphoid hematopathology.

Consistent with this pathology being an erythroid lineage proliferation, we noted a significant downregulation of CD45 on c-kit^+^, CD71^+^, and Ter119^+^ cells but not on B cells, T cells, or Mac1^+^ myeloid cells in the spleen and BM of diseased *Mx1*-*Cre*;*Brca1*^fl/fl^;*Trp53*^+/–^ mice (Figure 2F and data not shown). Under conditions of normal development, CD45 is expressed in all hematopoietic cells except terminally differentiated RBCs. In addition, aberrant downregulation of CD45 has been reported in leukemia cells in mice (26). The fact that we saw this aberrant loss of expression of CD45 selectively in the erythroid lineage further supports the erythroid origin of this hyperproliferation.

We confirmed that these phenotypes were not due to proliferative effects of pIpC-induced interferon by using Vav1-iCre to drive recombination in embryonic and adult HSCs and progenitors independent of pIpC (35). *Vavi*-*Cre*;*Brca1*^fl/fl^;*Trp53*^+/–^ mice also developed enlarged spleens with abnormal histopathology (Supplemental Figure 3, A–C), elevated WBC reads (Supplemental Figure 3D), and increased BM MEP frequencies (Supplemental Figure 3E).

### Erythroproliferative disease in Brca1/Trp53 deficient mice with the BRCA1^insC^ mutation.

**Figure 3:**
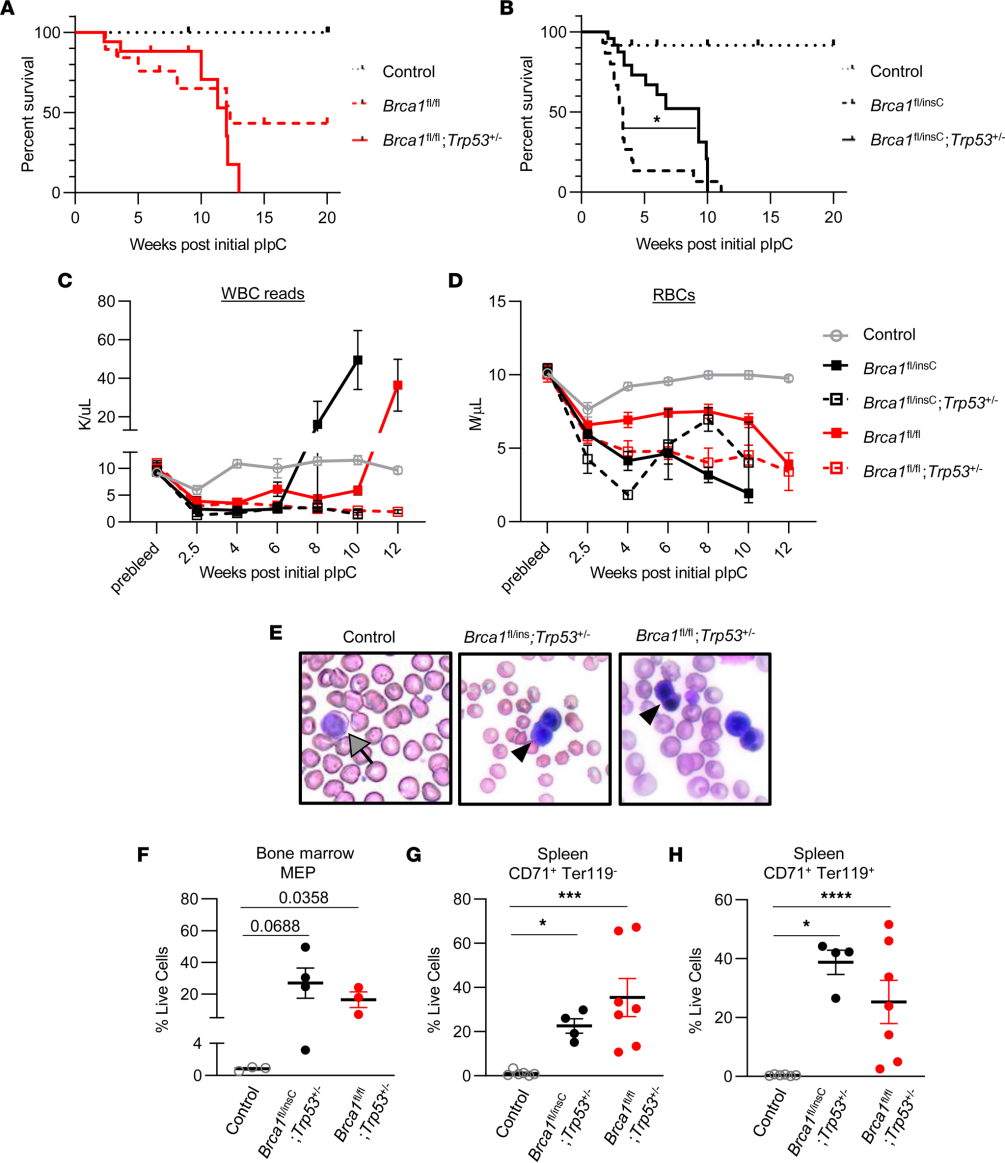
Oncogenic *BRCA1* 5382insC mutation accelerates the onset of *Brca1/Trp53* deficiency–associated hematological disease. (**A** and **B**) Kaplan-Meier curves of overall survival after initial pIpC treatment. Control (dotted black, *n* = 8), *Mx1-Cre;Brca1*^fl/fl^ (dashed red, *n* = 19), *Mx1-Cre;Brca1*^fl/fl^;*Trp53*^+/–^ (solid red, *n* = 17), *Mx1-Cre;Brca1*^fl/insC^ (dashed black, *n* = 15), and *Mx1-Cre;Brca1*^fl/insC^;*Trp53*^+/–^ (solid black, *n* = 24). (**C** and **D**) Peripheral blood WBC reads (indicative of erythroid blast cells) (**C**) and RBC (**D**) counts before (prebleed) and after initial pIpC treatment. Control (solid gray, *n* = 8), *Mx1-Cre;Brca1*^fl/insC^;*Trp53*^+/–^ (dashed black, *n* = 12), *Mx1-Cre;Brca1*^fl/insC^ (solid black, *n* = 9), *Mx1-Cre;Brca1*^fl/fl^;*Trp53*^+/–^ (dashed red, *n* = 16) and *Mx1-Cre;Brca1*^fl/fl^ (solid red, *n* = 5). (**E**) Wright-Giemsa–stained peripheral blood smears from age-matched control, *Mx1-Cre;Brca1*^fl/insC^;*Trp53*^+/–^, and *Mx1-Cre;Brca1*^fl/fl^;*Trp53*^+/–^ mice (gray arrow, normal lymphocyte; black arrowhead, erythroid progenitor). (**F**–**H**) Flow cytometric analysis of BM MEPs and spleen early (CD71^+^Ter119^–^) and mid (CD71^+^Ter119^+^) erythroid progenitors in control (*n* = 3-6), *Mx1-Cre;Brca1*^fl/insC^;*Trp53*^+/–^ (*n* = 4), and *Mx1-Cre;Brca1*^fl/fl^;*Trp53*^+/–^ (*n* = 7) mice. Statistical significance was assessed using log rank tests and 1-way ANOVA followed by Bonferroni correction (*P < 0.0167, ***P < 0.0003,****P < 0.00003). All mice carry *Mx1-Cre* except for controls, which were without *Mx1-Cre*.

The oncogenic *BRCA1*^insC^ mutation was evaluated in *Brca1/Trp53* deficiency to determine if it altered erythropoietic abnormalities as it had done with the pancytopenia in the setting of *Brca1* deficiency. We compared *Mx1*-*Cre*;*Brca1*^fl/insC^;*Trp53*^+/–^ mice to *Mx1*-*Cre*;*Brca1*^fl/fl^;*Trp53*^+/–^ mice and controls. These mice were in a 129S7/C57BL/6 mixed background. The median survival of *Mx1*-*Cre*;*Brca1*^fl/fl^ and *Mx1*-*Cre*;*Brca1*^fl/fl^;*Trp53*^+/–^ mice (12.3 and 12.0 weeks after initial pIpC induction, respectively) (Figure 3A) was comparable with that of prior experiments using mice in a pure C57BL/6 background (11.3 and 10.6 weeks, respectively). *Mx1*-*Cre*;*Brca1*^fl/insC^ mice showed earlier lethality with a median survival of 3.3 weeks (Figure 3B). Although added *Trp53* deficiency did not alter survival compared with simple *Brca1* nullizygosity, it did extend the survival of mice carrying *BRCA1*^insC^ allele (median survival *Mx1*-*Cre*;*Brca1*^fl/insC^;*Trp53*^+/–^, 6.7 weeks compared with *Mx1*-*Cre*;*Brca1*^fl/insC^; *P* = 0.0392) (Figure 3B). Similarly to *Mx1*-*Cre*;*Brca1*^fl/fl^;*Trp53*^+/–^ mice, *Mx1*-*Cre*;*Brca1*^fl/insC^;*Trp53*^+/–^ mice developed elevated WBC reads, indicative of the aberrant presence of erythroid blasts, following a period of low WBC reads (Figure 3C). This peripheral blood erythroblast phenotype was slightly more penetrant in *Mx1-Cre;Brca1*^fl/insC^*;Trp53^+/–^* mice (5 of 11, 45.45%) compared with *Mx1-Cre;Brca1*^fl/fl^*;Trp53^+/–^* mice (4 of 11, 36.4%) and occurred with significantly shorter latency (average time to elevated WBC reads, 8 versus 10 weeks, respectively; *P* = 0.0069) (Figure 3C and Supplemental Figure 4, A and B). Regardless of *Trp53* status, all *Brca1*-deficient mice displayed low RBC counts (Figure 3D). *Mx1*-*Cre*;*Brca1*^fl/insC^;*Trp53*^+/–^ mice also showed abnormal expansion of erythroid lineage cells in peripheral blood (Figure 3E), BM (Figure 3F), and spleen (Figure 3, G and H), as well as hepatosplenomegaly (Supplemental Figure 4, C and D). The expansions in the BM MEP compartments (~32-fold increase in *Mx1-Cre;Brca1*^fl/insC^*;Trp53^+/–^* and ~20 fold increase in *Mx1-Cre;Brca1*^fl/fl^*;Trp53^+/–^*) were not significant possibly due to small sample size. Enlarged spleens were effaced (Supplemental Figure 4, E–G), and liver showed mononuclear cell infiltration (Supplemental Figure 4, H–J). There were no elevations in B cell, T cell, or myeloid cell frequencies in the spleen (Supplemental Figure 4K). These phenotypes were not different from those of *Mx1*-*Cre*;*Brca1*^fl/fl^;*Trp53*^+/–^ mice. Therefore, although the disease developed significantly faster in the presence of the 5382insC allele compared with the null allele only, we conclude that both genotypes result in the same disease.

### Loss of heterozygosity (LOH) of Trp53 occurs in Brca1-deficient hematopoietic cells.

In patients with inherited *TP53* mutations and mouse models of cancer with heterozygous germline *Trp53* deficiency such as *Brca1*-deficient breast cancer models, the remaining WT allele is lost during tumorigenesis (23, 36–40). While interrogating the state of the genome in our *Mx1-Cre;Brca1*^fl/fl^*;Trp53^+/–^* mice with whole-exome sequencing (WES) of spleen and brain tissue, we determined if the normal *Trp53* allele in the *Trp53* heterozygous KO mice was intact. In enlarged *Mx1-Cre;Brca1*^fl/fl^*;Trp53^+/–^* spleens, we found there was loss of heterozygosity in the region deleted in the *Trp53-*null allele as compared with spleens from Cre^–^ control *Brca1*^fl/fl^*;Trp53^+/–^* mice (Supplemental Figure 5A). In contrast, brain tissue (where there was no expected Cre-mediated deletion of *Brca1*) from the same mice did not show LOH. Thus, the LOH of *Trp53* was dependent on the conditional deletion of *Brca1* in hematopoietic tissue. These data were confirmed with direct genotyping, as well (Supplemental Figure 5B). These data show that, although there initially was a *Trp53* heterozygous background, loss of the normal *Trp53* allele occurred following *Brca1* deletion and is part of the erythroproliferative process. Although we searched for other genetic alterations, including single nucleotide polymorphisms (SNPs), insertions and deletions (InDels), structural variants (SVs), and copy number variants (CNVs), no other relevant alterations were found.

### The erythroproliferative disease in Brca1- and Trp53-deficient mice is neoplastic.

Expansion of erythroid progenitor cells and impaired erythroid terminal differentiation are considered hallmarks of human acute erythroid leukemia (AEL) (41). Additionally, the erythroid phenotypes of our mice are reminiscent of those described in established murine models of acute erythroleukemia, including anemia, hepatosplenomegaly, infiltration of erythroblasts, and expansion of erythroid progenitors, suggesting that this, too, is a murine erythroleukemia (41).

**Figure 4:**
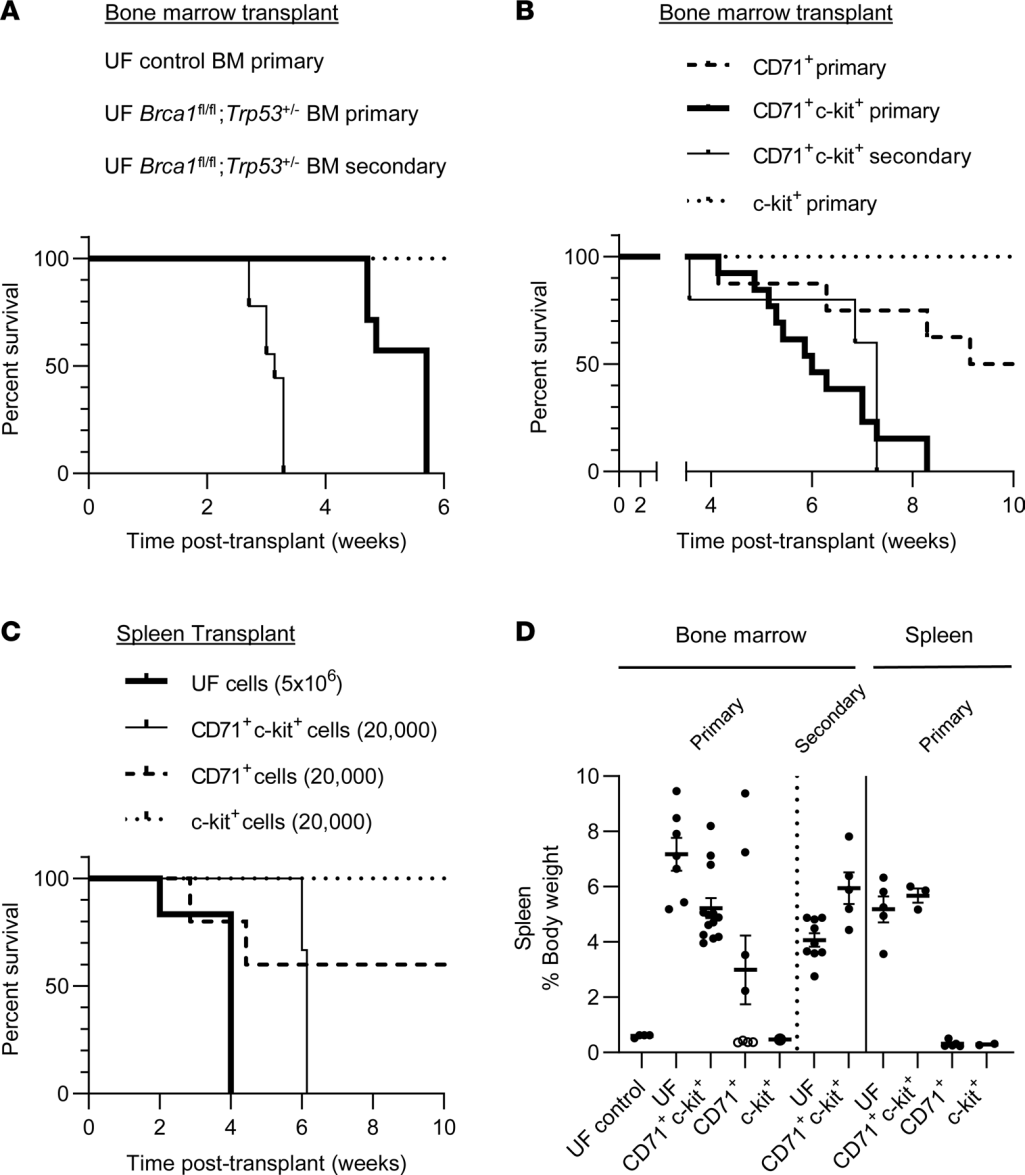
Leukemia of *Brca1* and *Trp53* double deficiency is transplantable through BM and spleen. (**A**) Kaplan-Meier curves of overall survival of primary (solid line, *n* = 7) and secondary (bold solid line, *n* = 9) recipients of unfractionated (UF) *Mx1-Cre;Brca1*^fl/fl^;*Trp53*^+/–^ BM. Recipients of UF control BM (dotted line, *n* = 4) did not show lethality. (**B**) Kaplan-Meier survival curves of primary recipients of CD71^+^ (dashed line, *n* = 8), CD71^+^c-kit^+^ (solid line, *n* = 13), c-kit^+^ (dotted line, *n* = 1), and secondary recipients of CD71^+^c-kit^+^ (bold solid line, *n* = 5) *Mx1-Cre;Brca1*^fl/fl^;*Trp53*^+/–^ BM cells. (**C**) Kaplan-Meier curves of overall survival of recipients of UF (bold solid line, *n* = 6), CD71^+^c-kit^+^ (solid line, *n* = 3), CD71^+^ (dashed line, *n* = 5), and c-kit^+^ (dotted line, *n* = 2) *Mx1-Cre;Brca1*^fl/fl^;*Trp53*^+/–^ spleen cells. (**D**) Terminal spleen weights of above recipient mice. Spleen weights of BM CD71^+^ recipient mice (**B**) that showed prolonged survival are marked by open circles. Data represent mean ± SEM.

To characterize the leukemogenicity of the *Brca1* and *Trp53* deficiency–associated hematopoietic neoplasm and to identify leukemia-initiating cells (LICs), we carried out transplantation experiments. Two million unfractionated (UF) BM cells from diseased *Mx1*-*Cre*;*Brca1*^fl/fl^;*Trp53*^+/–^ mice together with 200,000 WT congenic CD45.2 support cells were transplanted into lethally irradiated WT CD45.1 recipient mice. There was a requirement for support BM due to the necessity of Brca1 for the survival of HSCs (17). All recipients of UF BM developed the disease as indicated by splenomegaly, high WBC reads (indicative of erythroblasts in peripheral blood), and elevated erythroblast frequencies (CD71^+^c-kit^+^) in BM and spleen (Figure 4, A and D, and Supplemental Figure 6, A and B). Elevated erythroblast frequencies in recipient BM and spleen were comparable with those seen in the original *Mx1*-*Cre*;*Brca1*^fl/fl^;*Trp53*^+/–^ mice, with the highest frequency seen in spleen (Figure 2C and Supplemental Figure 6B). The disease could be serially transplanted into secondary recipients (Figure 4A). As has been reported previously in murine erythroleukemia models (25), the disease developed significantly faster in secondary recipients compared with primary recipients (median survival, primary versus secondary recipients, 5.7 versus 3.0 weeks after transplant, respectively; *P* = 0.0001). These transplant data demonstrate that the BM cells of the diseased mice have transformed, and they support the classification of this erythroblastic proliferation as a bona fide neoplastic disease.

To begin to identify the LICs, we sorted erythroblasts (CD71^+^c-kit^+^) from diseased *Mx1*-*Cre*;*Brca1*^fl/fl^;*Trp53*^+/–^ mice and tested their ability to propagate disease. In total, 20,000 CD71 and c-kit–double-positive (CD71^+^c-kit^+^) BM cells from diseased *Mx1*-*Cre*;*Brca1*^fl/fl^;*Trp53*^+/–^ mice initiated disease in all WT lethally irradiated recipients. Serial transplantation of disease with CD71^+^c-kit^+^ cells was also successful (Figure 4, B and D, and Supplemental Figure 6, A and B). Thus, this CD71c-kit^+^ population contains LICs. The capacity to propagate the disease through CD71^+^ immature erythroblasts has been reported in another mouse model of erythroleukemia (25). Tsuruta-Kishino et al. showed that a *Jak2* mutant, *Trp53*-deficient model of erythroid leukemia was able to use 50,000 sorted immature erythroid cells (CD71^+^Ter119^–^) from BM, along with 200,000 WT UF support BM, to consistently transmit the disease. Interestingly, in our model, we found that 20,000 CD71–single positive (CD71^+^) cells were less robust in transmitting disease compared with an equal number of CD71^+^c-kit^+^ cells, as evidenced by a prolonged survival of 10.1 weeks (Figure 4B) and only 50% disease penetrance (Figure 4D and Supplemental Figure 6, A and B). Due to the paucity of c-kit–single positive cells, we were only able to successfully transplant 1 mouse with c-kit^+^ cells, and this mouse did not display any disease phenotypes. In summary, we have found that the leukemia-initiating capacity in *Mx1*-*Cre*;*Brca1*^fl/fl^;*Trp53*^+/–^ mice is enriched in the CD71^+^c-kit^+^ cell population. The c-kit marker is expressed in leukemic blasts (26, 42), and here it helped select for a subpopulation of CD71^+^ cells with increased leukemia initiating capacity.

Similar to BM cells, spleen cells from diseased *Mx1*-*Cre*;*Brca1*^fl/fl^;*Trp53*^+/–^ mice transplanted the disease. Five million unsorted splenocytes with 200,000 WT support BM cells was readily transplanted into lethally irradiated WT recipients. As in BM transplantation, there was a necessity for support BM due to the HSC requirement for *Brca1*. All recipients died with splenomegaly, elevated WBC reads, anemia, and elevated erythroblasts by 4 weeks of age (Figure 4, C and D, and Supplemental Figure 6, C–E). As seen with BM, 20,000 CD71^+^c-kit^+^ cells from diseased *Mx1*-*Cre*;*Brca1*^fl/fl^;*Trp53*^+/–^ spleens were capable of transmitting the disease to lethally irradiated WT recipients with full penetrance. In contrast, none of the mice that received CD71–single positive cells or c-kit–single positive cells developed disease phenotypes at the completion of the experiment at 10 weeks after transplant (Figure 4, C and D, and Supplemental Figure 6, C–E). These transplant data show that the *Brca1*/*Trp53* deficiency–associated erythroid neoplasia is transplantable via CD71^+^c-kit^+^ cell populations, indicating that this population in the spleen contains LICs similar to those found in the BM.

The time it took for the disease to develop in recipients correlated with the number of cells used to transplant the disease. Average times to disease onset when 2.0 × 10^6^, 0.2 × 10^6^, or 0.02 × 10^6^ UF *Mx1*-*Cre*;*Brca1*^fl/fl^;*Trp53*^+/–^ spleen cells were used were 3.14, 4.07, and 5.57 weeks, respectively (Supplemental Figure 6F). Similarly, average time to disease was significantly prolonged from 4.7 weeks with 20,000 cells to 9.14 weeks with 1,000 cells when CD71^+^c-kit^+^ cells were used (Supplemental Figure 6G). Despite this difference, there were no differences in terminal disease state, as indicated by terminal WBC reads or degree of splenomegaly (Supplemental Figure 6, H and I). These transplant data show that hundreds of recipients with disease can be readily generated by transplanting spleen cells, thus eliminating the requirement to maintain large cohorts of syngeneic mice.

The transplantation experiments described to this point were done with cells isolated from *Mx1*-*Cre*;*Brca1*^fl/fl^;*Trp53*^+/–^ mice at an advanced state of disease when they have developed hepatosplenomegaly plus high levels of erythroblasts in peripheral blood (indicated by high WBC reads). However, we have found that the first indicator of disease is palpable splenomegaly, which occurs approximately 6 weeks after initial pIpC treatment. To investigate transplantability prior to full-blown disease, we transplanted lethally irradiated WT mice with 2 million UF BM or spleen cells from *Mx1*-*Cre*;*Brca1*^fl/fl^;*Trp53*^+/–^ mice 3.5 weeks or 6.5 weeks after initial pIpC treatment. Neither donor groups showed aberrant presence of erythroblasts in peripheral blood (no high WBC reads), but both did have anemia and modest splenomegaly (Supplemental Figure 7A). Neither BM nor spleen cells from the 3.5-week group transferred the disease into recipients (Supplemental Figure 7, B and D). In contrast, both BM and spleen from the 6.5-week donor group transferred the disease with full penetrance (Supplemental Figure 7, C and E). Spleen transplant recipients developed the disease significantly faster than BM transplant recipients (spleen versus BM average time to disease 6.3 versus 8.7 weeks after transplant, *P* < 0.05) (Supplemental Figure 7C); therefore, spleen is more suitable for rapid disease propagation in recipient cohorts.

### Olaparib attenuates the erythroproliferative phenotypes in Brca1/Trp53-deficient mice.

**Figure 5:**
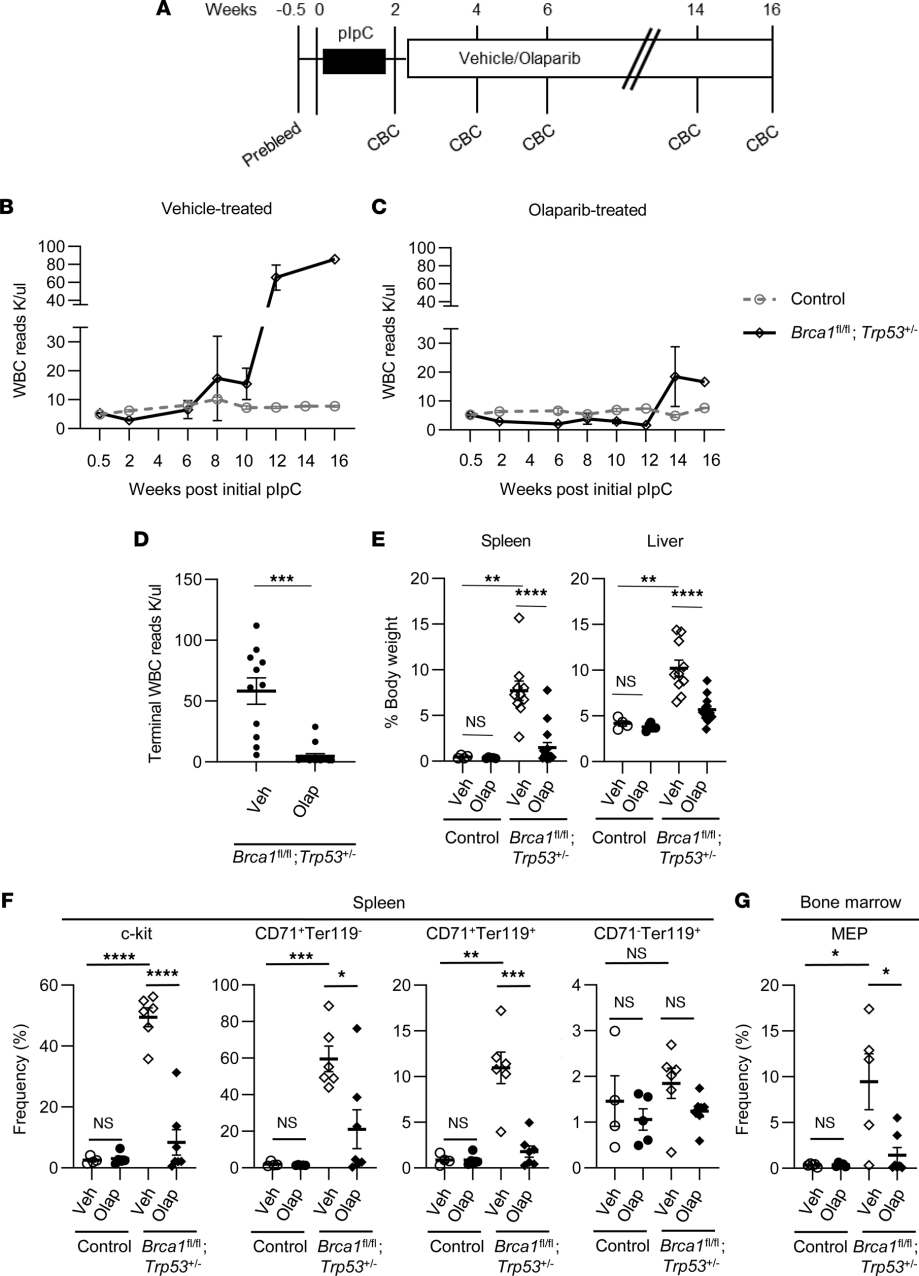
PARP inhibitor olaparib attenuates *Brca1/Trp53* deficiency–associated hematopoietic phenotypes. (**A**) Schematic representation of the olaparib treatment schedule. (**B** and **C**) Average WBC reads (erythroid blast cells) of vehicle- or olaparib-treated control and *Mx1-Cre;Brca1*^fl/fl^;*Trp53*^+/–^ mice. Vehicle, control (*n* = 8); vehicle, *Mx1-Cre;Brca1*^fl/fl^;*Trp53*^+/–^ (*n* = 13); olaparib, control (*n* = 9); olaparib, *Mx1-Cre;Brca1*^fl/fl^;*Trp53*^+/–^ (*n* = 15). (**D**) Terminal WBC reads (erythroid blast cells) in vehicle-treated (*n* = 13) or olaparib-treated (*n* = 15) *Mx1-Cre;Brca1*^fl/fl^; *Trp53*^+/–^ mice. (**E**) Terminal spleen and liver weights of vehicle- or olaparib-treated control and *Mx1-Cre;Brca1*^fl/fl^;*Trp53*^+/–^ mice. Control, vehicle (*n* = 4); Control,olaparib (*n* = 5); *Mx1-Cre;Brca1*^fl/fl^; *Trp53*^+/–^, vehicle (*n* = 10); *Mx1-Cre;Brca1*^fl/fl^;*Trp53*^+/–^, olaparib (*n* = 15). (**F** and **G**) Flow cytometric analysis of spleen (**F**) and BM (**G**) show decreased c-kit^+^, CD71^+^ (early Ter119^–^ and mid Ter119^+^) erythrocyte and megakaryocyte/erythroid progenitor (MEP) frequencies in olaparib-treated *Mx1-Cre;Brca1*^fl/fl^;*Trp53*^+/^ mice compared with those treated with vehicle. No change in CD71^–^Ter119^+^ late erythrocytes (control, *n* = 4–5; *Mx1-Cre;Brca1*^fl/fl^;*Trp53*^+/–^, *n* = 6–7). Data represent mean ± SEM. Statistical significance was assessed using a 2-tailed Student’s *t* test (*P < 0.05, **P < 0.01, ***P < 0.001,****P < 0.0001) (**D**) or 1-way ANOVA followed by Bonferroni correction (*P < 0.0125, **P < 0.0025, ***P < 0.0002, ****P < 00002) (**E**–**G**). Controls were without *Mx1-Cre*, all other mice carry *Mx1-Cre*.

Olaparib is a PARP inhibitor approved for use in *BRCA*-mutated ovarian, prostate, and pancreatic cancers (8). Olaparib treatment delays mammary tumor development in *Brca1/Trp53*-deficient mice (43). We hypothesized that olaparib may similarly inhibit the *Brca1* and *Trp53* deficiency–associated erythroid neoplasia. After the final pIpC mediated induction of Cre, we initiated olaparib treatment (Figure 5A). Mice received i.p. injections of either olaparib or vehicle every other day and were evaluated when moribund. A majority (90.9%) of vehicle-treated *Mx1*-*Cre*;*Brca1*^fl/fl^;*Trp53*^+/–^ mice developed the expected elevated WBC counts (average 68.4 K/μL) indicative of disease (Figure 5, B and D). In contrast, of the olaparib-treated *Mx1*-*Cre*;*Brca1*^fl/fl^;*Trp53*^+/–^ mice, only 2 (13.3%) developed elevated WBC counts (Figure 5, C and D), and these elevations were modest (16.6 K/μL and 28.8 K/μL). Olaparib was also able to abrogate the hepatosplenomegaly associated with *Brca1/Trp53* deficiency (Figure 5E). Olaparib was nontoxic, as control mice showed no changes in WBC reads, spleen and liver weights, or viability. These data show that treatment with olaparib prior to disease development significantly prevented the highly penetrant neoplastic phenotypes observed in untreated *Mx1*-*Cre*;*Brca1*^fl/fl^;*Trp53*^+/–^ mice.

In addition to successful suppression of gross disease in hematopoietic tissues by olaparib treatment, flow cytometric analysis of hematopoietic tissues showed absence of the disease-associated expansion of erythroid populations in spleen (Figure 5F), BM (Figure 5G), and peripheral blood (data not shown). The elevated frequencies of c-kit^+^ cells and CD71^+^ early/mid erythroid progenitors in the spleen were reduced to control levels. In contrast, the frequencies of CD71^–^Ter119^+^ late erythroid progenitors (that were not elevated in diseased tissue) were not changed by olaparib treatment (Figure 5F). Similar to immature erythroblasts, the increased frequencies of MEPs in BM were significantly reduced by olaparib treatment (2.9-fold, *P* = 0.0146) (Figure 5G). Genetic analysis of spleens from olaparib- or vehicle-treated animals showed that the cells diminished by olaparib treatment were, as expected, the *Brca1*- and *Trp53*-deficient cells. The unrecombined floxed *Brca1* allele and the WT *Trp53* allele were predominant in olaparib-treated spleens compared with vehicle-treated spleens (Supplemental Figure 8, A–C). In contrast, no differences were found in our genetic analysis of brain tissue when comparing olaparib and vehicle (Supplemental Figure 8, D–F).

*Vavi-Cre Brca1*^fl/fl^ mice were also sensitive to olaparib (Supplemental Figure 8). In this case, olaparib treatment further reduced the already low peripheral blood counts of *Vav-Cre Brca1*^fl/fl^ mice (WBCs, 2.55-fold, *P* = 0.0015; RBCs, 2.64-fold, *P* = 0.0001) compared with no effect on WBCs and a mild 1.16× effect on RBCs in controls (Supplemental Figure 8, G and H). Spleen weights were also significantly decreased (2.24-fold, *P* = 0.0055) by olaparib treatment in *Vav-Cre Brca1*^fl/fl^ mice but not in controls (Supplemental Figure 8I). In summary, these data demonstrate that pretreatment with olaparib can prevent the development of *Brca1/Trp53* double deficiency–associated erythroproliferative neoplasm through selective elimination of *Brca1* deficient cells.

These data indicate that the hematologic disease in double *Brca1* and *Trp53*–deficient mice is a rapidly fatal, olaparib-sensitive erythroid neoplasia. The olaparib sensitivity, the ability to transplant the disease to multiple recipients, and the ability to monitor disease through peripheral blood counts all suggest that this *Brca1/Trp53* deficiency syngenic immune-replete mouse model will be useful for achieving rapid in vivo screens for new anticancer agents against *BRCA1*- or *TP53*-deficient tumors.

## Discussion

In this report and in our prior publication (17), we show that hematopoietic *Brca1* deficiency in mice leads to BM failure. Expanding on these discoveries, we now report that *Brca1* deficiency, when combined with *Trp53* deficiency, leads to the development of a murine erythroid leukemia that is rapid onset, aggressive, and ultimately fatal. It is established that BRCA1’s role in HR-mediated DNA DSB repair is important in the maintenance of genomic stability and tumor suppression (10, 44). We and others have previously shown that, as would be expected, deletion of *Brca1* in the hematopoietic cells leads to increased DNA damage and genomic instability (17, 19). *Brca1* deficiency and associated defects in DNA repair are not tolerated in the hematopoietic system and leads to BM failure and lethality.

These data are consistent with the “transform or die” hypothesis (17), where Brca1 loss is lethal to hematopoietic cells unless there are secondary genetic changes, such as *Trp53* loss of function, which can drive neoplastic transformation. Although, in our model, we started with a *Trp53* heterozygous background, the WT *Trp53* allele was lost in enlarged *Mx1-Cre*;*Brca1*^fl/fl^;*Trp53*^+/–^ spleens of leukemic mice (Supplemental Figure 5). As seen with other models, loss of heterozygosity of *Trp53* could be a necessary early step in the development of leukemia. In a *JAK2*^V617F^ mouse model of erythroleukemia, *Trp53* nullizygosity was essential for disease development; in a *Trp53* heterozygous background, *JAK2*^V617F^ mice did not develop the erythroleukemia of *JAK2*^V617F^;*Trp53*^–/–^ mice but were similar to *JAK2*^V617F^ mice (25). Unlike with the *JAK2*^V617F^ mutation, *Brca1* deficiency created a background permissive for the loss of the normal *Trp53* allele to initiate tumorigenesis, consistent with BRCA1 functions in maintaining genome integrity (44). We originally thought that *Brca1*- and *Trp53*-deficient cells would accumulate additional mutations that support proliferation and self-renewal but didn’t find additional meaningful changes. It is possible that alterations that effect these processes are present in the noncoding regions not covered by WES. Additionally, WES has low sensitivity for certain SVs such as rearrangements. Therefore, our analysis may have missed relevant alterations.

A single spontaneous case of erythroleukemia has been reported in a *Mx1-Cre*–driven *Brca1*-deficient mouse model (19). However, this erythroleukemia developed at a low frequency (1 of 13 mice) and without mutations in *Trp53*, suggesting that this erynthroleukemia is probably of different etiology compared with ours and suggesting that *Brca1* deficiency–associated erythroleukemia can develop via other mechanisms independent of *Trp53* deficiency.

In this study, we show that the *Brca1/Trp53* double deficiency–associated hematopoietic neoplasia can be prevented with the PARP inhibitor olaparib. Prior in vivo mouse studies have shown that *Brca1/Trp53* deficiency–driven mammary tumors are sensitive to PARP inhibition (7, 43). PARP inhibitor sensitivity suggests that leukemia cells share the synthetic lethality of combined *Brca1* deficiency and PARP inhibition demonstrated in mice with *Brca1/Trp53*-deficient mammary tumors. Although the *Brca1/Trp53*-deficient breast tumor models are important in that they recapitulate features of human breast cancer (39, 40, 45–52), they are inefficient for testing drug responses due to long tumor latency (>6–12 months). In addition, the BLG-Cre system requires repeated pregnancies to induce recombination of the *Brca1* allele, which makes the system cumbersome. Our *Brca1/Trp53*-deficient hematopoietic model has advantages over existing *Brca1* models and could provide a novel preclinical platform to test putative drugs targeting *Brca1*-deficient (or *Trp53*-deficient) cancers in vivo. The ability to transplant the disease to multiple recipients and generate large cohorts of syngenic mice, the ability to monitor disease through peripheral blood counts, the rapid disease onset, and the high penetrance all suggest that this *Brca1/Trp53*-deficiency mouse model will be useful for achieving rapid in vivo drug screening.

Whether mouse models can be used to understand the pathogenicity of human *BRCA1* mutations has not been extensively explored. Using our humanized *BRCA1*^insC^ allele, we have established the pathogenicity of this mutation in hematopoietic tissue. We have preliminary data suggesting that this mutation is pathogenic in mammary tissue, as well. Similarly to *BLG-cre*;*Brca1*^fl/fl^;*Trp53*^+/–^ mice, *BLG-cre*;*Brca1*^fl/insC^;*Trp53*^+/–^ mice developed mammary tumors with characteristics of basal-like breast adenocarcinoma (49, 51) with near-absent mRNA expression of estrogen and progesterone receptors, as has been reported before for *Brca1*-null, *Trp53*-deficient mammary tumors (Supplemental Figure 9 and data not shown, respectively). These data show, for the first time to our knowledge, that the human *BRCA1*^insC^ mutation causes breast cancer when in combination with *Trp53* deficiency.

A previous study had shown that a mouse *Brca1* allele with a premature stop codon at an analogous human 5382 position predisposes mice to breast cancer (53). However, in humans, the 5382insC mutation does not lead to a simple truncation; instead, it is a frameshift that replaces the last 69 amino acids with a potentially novel peptide sequence at the C-terminus. Whereas this mouse *Brca1* 5382stop truncation resulted in loss of expression of the BRCA1 protein, the human 5382insC mutant protein is not lost (54, 55). Therefore, this mouse *Brca1* mutant model may not reflect the tumorigenic behavior of an expressed human *BRCA1*^insC^ protein product. In contrast to this allele, our *BRCA1*^insC^ allele leads to the expression of a detectable, stable protein in mouse cells (17).

It is interesting that the *Brca1*-null phenotype is exacerbated by the presence of the *BRCA1*^5382insC^ alleles, although it doesn’t show dominant negative effects in a *Brca1* WT background (17). This suggests that the mutant protein is detrimental only in the absence of WT Brca1. Multiple activities of BRCA1, including those of DNA repair, involve its interaction with other proteins. The *BRCA1*^insC^ mutation results in the expression of a truncated BRCA1 missing the C-terminal BRCT repeat that interacts with phosphopeptides and promotes HR, which is necessary for tumor suppression (56). The greater pathogenicity of the *BRCA1*^insC^ allele suggests that the mutant protein effects cellular functions in other ways, as well. The *BRCA1*^insC^ mutant protein retains a large part of the WT sequence and may, therefore, retain WT properties. It may bind and sequester key proteins and DNA sequences, disrupting their proper function. Alternatively or additionally, the mutation may lead to changes in its structure that interfere with its normal function. For example, studies in the breast cancer cell line HCC1937 that carries the *BRCA1*^insC^ mutation have shown that structural changes near the N-terminal RING domain lead to changes in posttranslational modifications under conditions of cell stress (57). The distinct effects of the *BRCA1*^insC^ mutation in hematopoietic cells, in comparison with the *Brca1* null mutation, can only be understood with more research.

Our analysis of the *BRCA1*^insC^ mutation shows that our hematopoietic model could serve as a genetic model to asses the pathogenicity of *BRCA1* variants. In humans, *BRCA1*^insC^ mutation is one of the relatively common inherited mutations that predispose to hereditary cancer syndromes; therefore, it is well established as a pathogenic mutation. With the increase in sequencing, many other less common variants/mutations of *BRCA1* have been identified (58). A significant number of these variants are classified as variants of unknown significance (VUS), since their clinical significance, whether pathogenic or benign, cannot be determined due to the lack of sufficient evidence. Many attempts have been made to develop in vitro functional assays to determine the pathogenicity of the VUSs (59), but they don’t address the complexity of in vivo biology. Similar to improved in vivo drug screening, assessing *BRCA1* variants in the murine mammary tumor system is a laborious task, and our hematopoietic *Brca1/Trp53* leukemia model could provide an alternative model.

In future studies, it will be of interest to carry out molecular and genetic experiments to understand the mechanisms of how *Brca1* deficiency–associated genomic instability and other defects lead to the hematopoietic phenotypes. For example, TP53BP1 is a DNA damage response protein that can reverse phenotypes of *Brca1* mutant cells (59). TP53BP1 promotes nonhomologous end joining (NHEJ) repair and suppresses HR (60). TP53BP1 loss has been shown to partially restore HR defects and reduce genomic instability seen in *Brca1*-deficient cells (61). We would expect that the codeletion of *Tp53bp1* would at least partially rescue the BM failure associated with *Brca1* deletion. Such a rescue will confirm that it is the *Brca1* deficiency–associated HR defects that lead to BM failure. Restoration of HR and alleviation of genomic instability would create a milieu less permissible to tumorigenesis and reduce the likelihood that LOH of *Trp53,* which we assume is an early driver of leukemic transformation, would occur.

In this report and in our prior publication (17), we show that hematopoietic *Brca1* deficiency in mice leads to pancytopenia. Expanding on these discoveries, we now report that *Brca1* deficiency, when combined with *Trp53* deficiency, leads to the development of a murine erythroid leukemia that has a rapid onset, is aggressive, and is ultimately fatal.

## Methods

### Mice.

The *Brca1*^fl^ (*Brca1*^fl22–24^) (49), *Brca1*^insC^ (17), *Mx1-Cre* (28), *Vav1-iCre* (35), and *BLG-cre* (49) alleles have been described previously. The generation and characterization of the *BRCA1*^insC^ allele was is described in detail in our prior publication (17). Briefly, we first generated mice that had WT human *BRCA1* cDNA knocked in to the mouse *Brca1* locus (*BRCA1*^WT^). These mice expressed WT human *BRCA1* instead of mouse *Brca1*. This WT human *BRCA1* allele was designed in such a way that, upon Cre-mediated recombination, it expressed the 5382insC mutant version (*BRCA1*^insC^) instead of the WT *BRCA1*. Mice with *BRCA1*^insC^ allele in the germline was generated by crossing mice carrying the *BRCA1*^WT^ allele to CMV-Cre deletor mice (62). The *BRCA1^insC^* allele has since been maintained through breeding in our colony. All animals were generated on a C57BL/6 pure background except for the mice in Figure 3; Supplemental Figures 2–4; Supplemental Figure 8, G–I; and Supplemental Figure 9, which are in a BL6/129S mixed background.

*Mx1-Cre* recombination was induced by administering 8 i.p. pIpC (GE Healthcare) injections (10mg/kg), every other day. Following pIpC injections, mice were monitored regularly for evidence of disease and were sacrificed when moribund. Expression of *BLG-cre* recombinase was induced in female mice undergoing, on average, 4 pregnancies. Animals were monitored for tumor development and sacrificed for analysis when maximum tumor size allowable was reached (~1/10 body weight) or when the health of the mouse was otherwise compromised.

Mice were weaned around 4 weeks of age and genotyped from tail snips using real-time PCR assays designed by Transnetyx. All experimental animals were housed in the Unit for Laboratory Animal Medicine at the UT Southwestern Medical Center under specific pathogen–free conditions. Experimental procedures were conducted after approval by the UT Southwestern IACUC (APN 2017-102119).

### Histology.

Tissue samples were fixed in 4% paraformaldehyde/PBS (Thermo Fisher Scientific); bones (femur and tibia) were fixed in Cal-Rite decalcifying solution (Thermo Fisher Scientific). The HistoPathology Core at UT Southwestern Medical Center then performed paraffin processing and embedding, mounted 5 μm–thick sections on slides, and stained the slides with H&E for morphological analysis. Blood smears were prepared for H&E staining following manufacturer’s directions (Sigma-Aldrich).

### Hematopoietic analysis.

For flow experiments, BM and spleen single-cell suspensions were created as described (63). For HSC and progenitor analysis, cells were incubated with biotinylated lineage specific antibodies (CD3ε, CD4, CD5, CD8a, CD45R, CD11b, Gr-1, Ter119) and fluorophore-conjugated antibodies against progenitor surface markers (Sca1, c-kit, CD16/32, CD34, CD48, CD150). PE-CF594 streptavidin was used to identify lineage-positive cells and DAPI (MilliporeSigma) to exclude dead cells. For lineage analysis, samples were incubated with combinations of fluorophore-conjugated antibodies to the following cell surface markers: CD117, CD3ε, CD4, CD8a, CD45, CD45R, CD11b, Gr-1, Ter119, and CD71. Antibodies used in flow cytometric analysis are included in Supplemental Table 1.

Flow samples were analyzed using the FACS Canto RUO analyzer (BD Biosciences). Gating schemes for hematopoietic progenitors were performed as previously described (17, 63, 64) using fluorescence minus 1 control. Complete peripheral blood counts were assessed using the HemaVet HV950 with MULTI-TROL Mouse as an equilibration control (Drew Scientific). Blood smear slides were stained using Hemacolor Stain Set (Harleco) according to manufacturer’s instructions.

### WES.

Sample preparation, sequencing, and bioinformatics analysis was performed by CD Genomics. WES data from each spleen were compared with respective paired brain tissue. DNA was extracted using the Qiagen DNeasy kit. Quantity of DNA was measured by picogreen method using Victor X2 fluorometry. Integrity of DNA were checked by Agilent genomic DNA screentape, offering a numeric measurement DNA integrity number (DIN). Sequencing libraries were generated using Agilent SureSelectXT Kit (Agilent Technologies) following manufacturer’s recommendations, and index codes were added to attribute sequences to each sample. Read length for paired-end reads was 151 bp. Barcoded WES libraries were sequenced on Illumina-based platform. Average depth was 136×. Paired-end clean reads were aligned to mouse reference genome GRCm38.p6 using Burrows-Wheeler Aligner (BWA) software (65). Picard was used to mark duplicates. GATK Best Practices were followed for preprocessing BAM files (66). GATK and SAMtools were used for variant calling. SnpEff program was used to examine structural changes. Sequences were aligned and graphically visualized using the Integrative Genomic Viewer (IGV) (http://software.broadinstitute.org/software/igv/). WES data were deposited in the Sequence Read Archive (SRA) database (accession no. PRJNA887455).

### Drug treatment.

Olaparib (AZD2281, LC Laboratories) was dissolved in DMSO (Thermo Fisher Scientific) and stored as a 50 mg/mL stock. Prior to use, the olaparib stock was diluted with 10% 2-hydroxyl-propyl-β-cyclodextrine/PBS (Fisher Scientific) in a 1:10 ratio (v/v). Vehicle preparation was the same minus olaparib. All mice began treatment 2.5 weeks after the final pIpC injection. Mice received vehicle or olaparib (50 mg/kg) i.p. injections every other day.

### BM transplantation.

BM transplantations were done as previously described (17). Adult recipient mice (CD45.1) were administered a minimum lethal dose of radiation using an XRAD 320 x-ray irradiator (Precision X-Ray) to deliver 2 doses of approximately 540 rad (1,080 rad in total) at least 3 hours apart. Cells were injected into the retro-orbital venous sinus of anesthetized recipients. Blood was obtained from the submandibular plexus of recipient mice at the indicated time points after transplantation. RBCs were lysed with ammonium chloride potassium buffer.

### Statistics.

Data are displayed as mean ± SEM. Statistical significance between 2 groups was assessed using the 2-tailed Student’s *t* test. Statistical significance between multiple groups was determined by 1-way ANOVA followed by Bonferroni corrected post hoc comparisons. To perform Bonferroni correction, statistical comparisons were made using unpaired 2-tailed Student’s *t* tests, and the critical *P* value (α) was divided by the number of comparisons being made. Log-rank test was used for survival analysis. A χ^2^ analysis was assessed with the Fisher’s exact test. *P* values less than 0.05 were considered significant.

### Study approval.

The present studies in mice were reviewed and approval by the UT Southwestern IACUC, UT Southwestern Medical Center (APN 2017-102119).

## Author contributions

TSR and VEM initiated the study, and GLP, KM, SRH, RW, and TSR designed/interpreted the experiments. GLP, KM, SRH, and RW performed experiments and collected the data.

## Supplementary Material

Supplemental data

## Version 1 11/08/2022

In-Press Preview

## Version 2 12/22/2022

Electronic publication
