# Supplementary material for: BRCA1 and TP53 codeficiency causes a PARP inhibitor–sensitive erythroproliferative neoplasm
Source: JCI Insight. 2022 Dec 22;7(24):e158257. doi: 10.1172/jci.insight.158257 (PMC9869974; doi:10.1172/jci.insight.158257)
Supplement: Supplemental data [file jciinsight-7-158257-s070.pdf]

Supplemental Figure 1

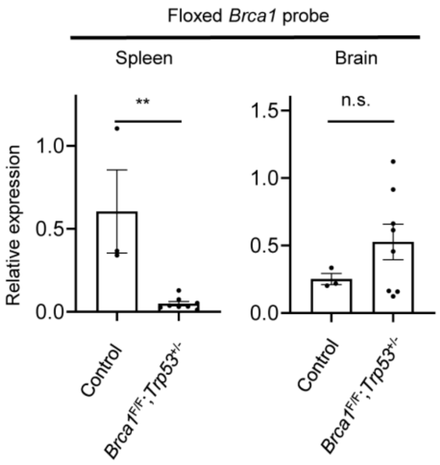

Supplemental Figure 1. Recombination of the *Brca1* floxed allele in hematopoietic tissue following pIpC treatment. RT-PCR genotyping analysis of *Brca1* allele status in spleen and brain of control *Brca1*$^{F/F}$;*Trp53*$^{+/-}$ (n=3) and diseased *Mx1-Cre*;*Brca1*$^{F/F}$;*Trp53*$^{+/-}$ (n=8) mice. Values represent mean ± SEM. Statistical significance was assessed using a two-tailed Student's t test (*p < 0.05, **p < 0.01, ***p < 0.001,****p < 0.0001). All mice carry *Mx1-Cre* except controls.

Supplemental Figure 2 – related to Figures 1 and 2

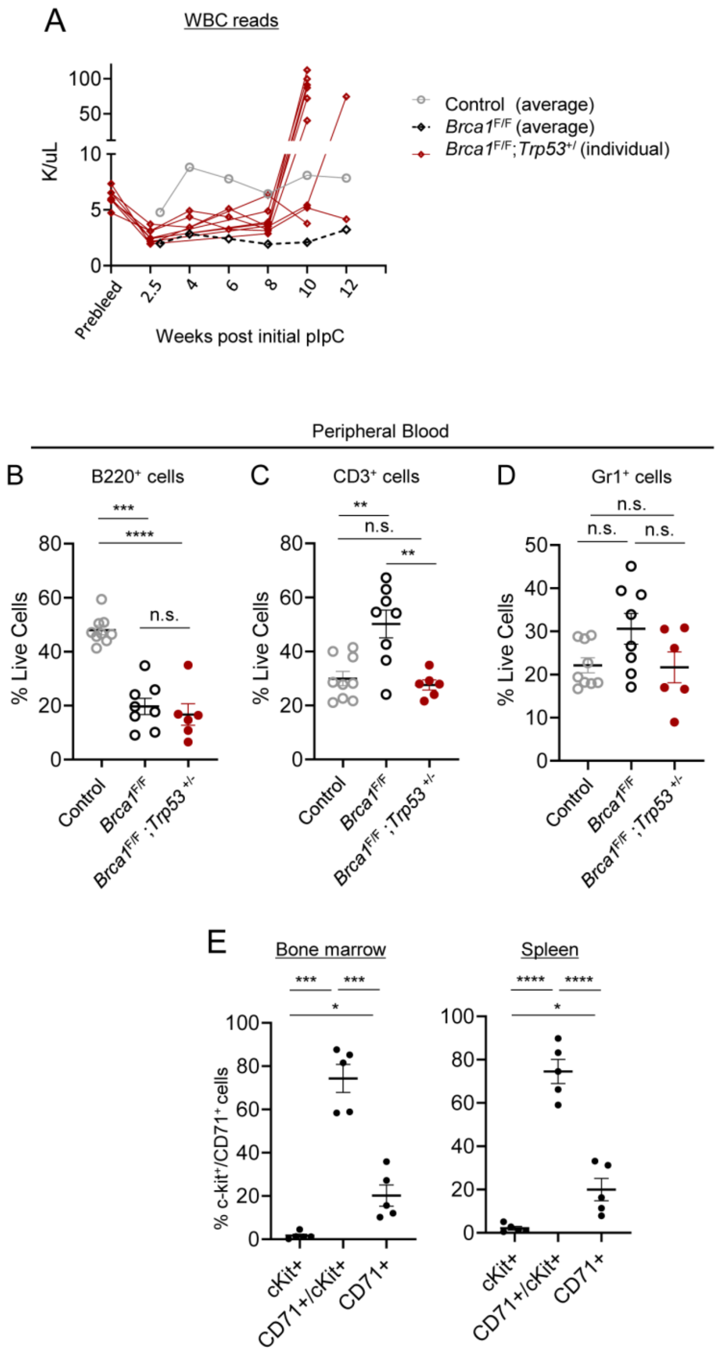

Supplemental Figure 2, related to Figures 1 and 2. Expansion of erythroid lineage in *Mx1-Cre*;*Brca1*$^{F/F}$;*Trp53*$^{+/-}$ mice. (A) Individual peripheral blood white blood cell (WBC) reads* of *Mx1-Cre;Brca1*$^{F/F}$;*Trp53*$^{+/-}$ mice (n=9) and average WBC reads* of control (n=13) and *Mx1-Cre;Brca1*$^{F/F}$ (n=11) mice before (prebleed) and after pIpC treatment. * Indicate erythroid blast cells. **(B-D)** No increase of B220^{+} B cell (B), CD3^{+} T cell (C), or Gr1^{+} cell (D) frequencies in peripheral blood of diseased *Mx1-Cre;Brca1*$^{F/F}$;*Trp53*$^{+/-}$ mice (n=9) compared to control (n=8) or *Mx1-Cre;Brca1*$^{F/F}$(n=6) mice. **(E)** Frequencies of c-kit^{+}, CD71^{+}/c-kit^{+}, and CD71^{+} cells in bone marrow and spleen in diseased *Mx1-Cre;Brca1*$^{F/F}$;*Trp53*$^{+/-}$ mice (n=5) show the high abundance of CD71 and c-kit double positive cells. Values represent mean ±SEM. Statistical significance was assessed using one-way ANOVA followed by Bonferroni correction (*p < 0.0167, **p < 0.003, ***p < 0.0003,****p < 0.00003). Controls were without *Mx1-Cre*, all other mice carry *Mx1-Cre*.

Supplemental Figure 3

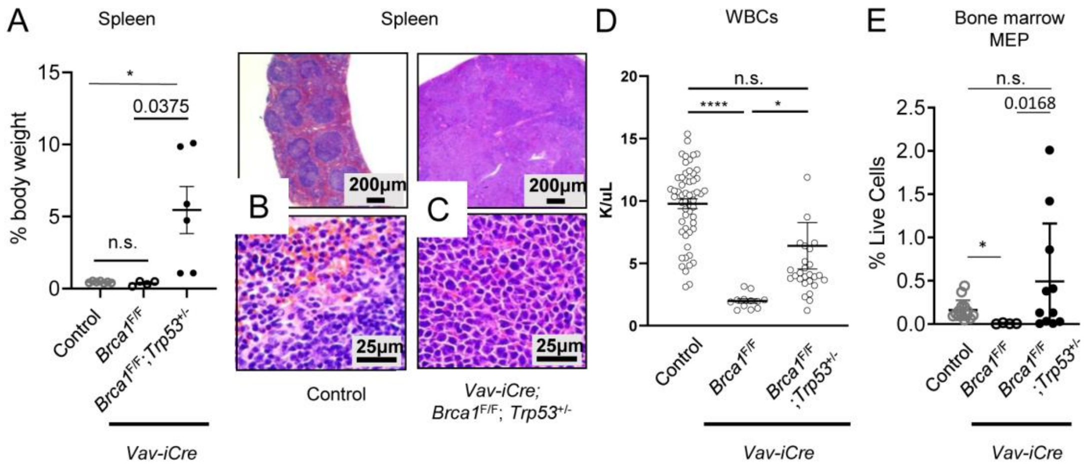

Supplemental Figure 3. *Vav1-iCre; Brca1*$^{F/F}$;*Trp53*$^{+/-}$ mice recapitulate hematopoietic phenotypes of Mx1-Cre driven *Brca1*/*Trp53* deficiency. (A) *Vav1-iCre;Brca1*$^{F/F}$;*Trp53*$^{+/-}$ mice develop pIpC-independent splenomegaly. Increased spleen weights of *Vav1-iCre;Brca1*$^{F/F}$;*Trp53*$^{+/-}$ (n=6) mice compared to control (n=7) and *Vav1-iCre;Brca1*$^{F/F}$ (n=4) mice. **(B-C)** H&E stains of spleen sections show that compared to controls (B), *Vav1-iCre;Brca1*$^{F/F}$;*Trp53*$^{+/-}$ spleens (C) are effaced with monomorphic cells. **(D-E)** Compared to cytopenic *Vav1-iCre*;*Brca1*$^{F/F}$ mice, *Vav1-iCre*;*Brca1*$^{F/F}$;*Trp53*$^{+/-}$ mice have higher white blood cell (WBC) reads (D) and bone marrow megakaryocyte/erythroid progenitor (MEP)(E) frequencies. WBC and flow cytometry numbers: Control (n=20), *Vav1-iCre*;*Brca1*$^{F/F}$ (n=11), *Vav1-iCre*;*Brca1*$^{F/F}$;*Trp53*$^{+/-}$ (n=18). Values represent means ±SEM. Statistical significance was assessed using one-way ANOVA followed by Bonferroni correction (*p<0.0167, **p<0.003, ***p<0.0003,****p<0.00003). Controls were either wildtype or without *Vav1-iCre*.

Supplemental Figure 4 – related to Figure 3

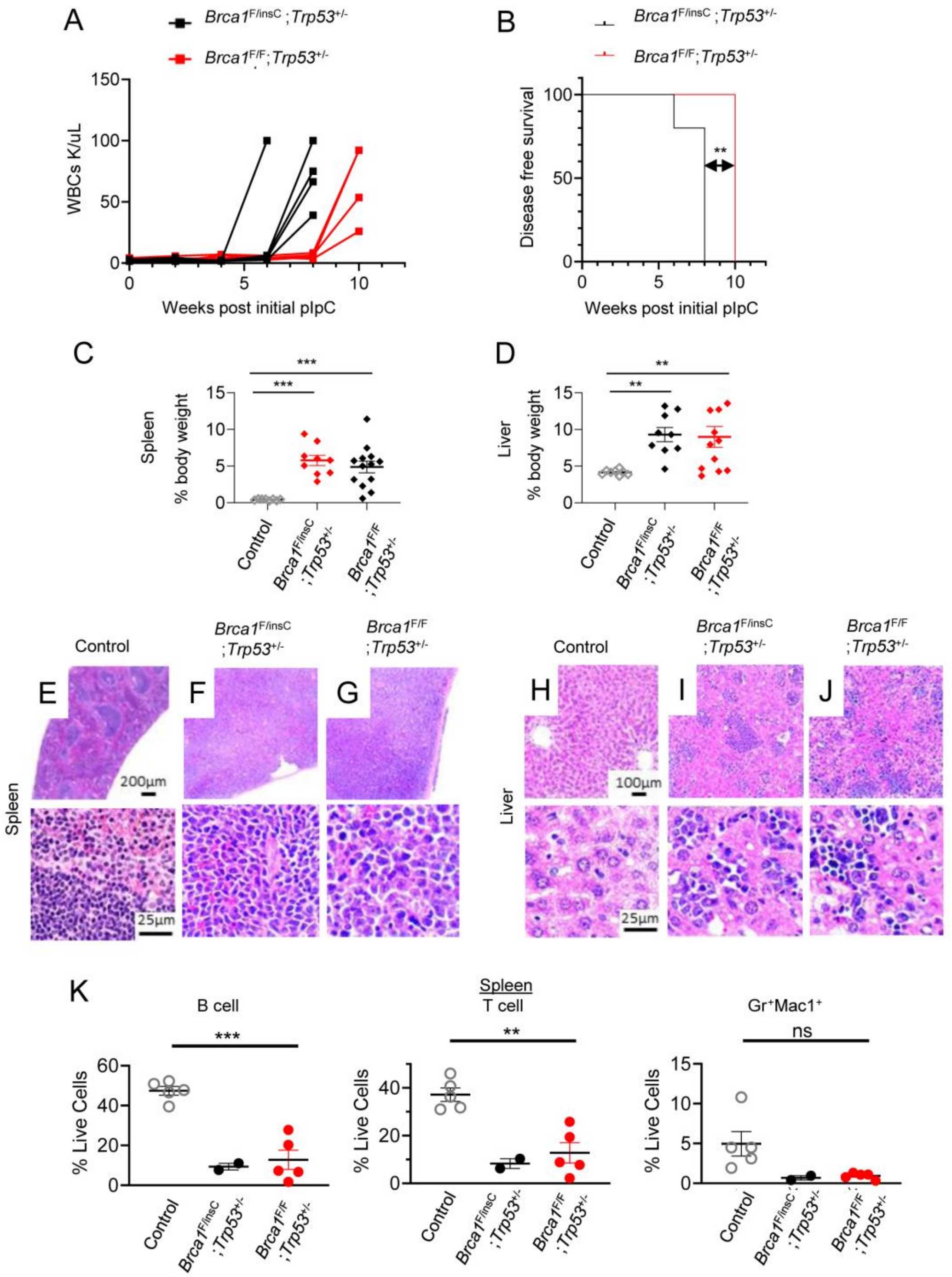

Supplemental Figure 4, related to Figure 3. *Mx1-Cre;Brca1*$^{F/insC}$;*Trp53*$^{+/-}$ mice develop an erythroproliferative disease similar to that of *Mx1-Cre;Brca1*$^{F/F}$;*Trp53*$^{+/-}$ mice. (A) Individual mouse white blood cell (WBC) reads* show that *Mx1-Cre*;*Brca1*$^{F/insC}$;*Trp53*$^{+/-}$ mice (black, n=5) develop high WBC reads* earlier than *Mx1-Cre*;*Brca1*$^{F/F}$;*Trp53*$^{+/-}$ mice (red, n=4). **(B)** Time to elevated WBC read* in *Mx1-Cre;Brca1*$^{F/insC}$;*Trp53*$^{+/-}$ (black, n=5) and *Mx1-Cre;Brca1*$^{F/F}$;*Trp53*$^{+/-}$ (red, n=4) mice. **(C-D)** Spleen (C) and liver (D) weights 8-12 weeks post initial pIpC treatment. The spleens of *Mx1-Cre;Brca1*$^{F/insC}$*;Trp53*$^{+/-}$ and *Mx1-Cre;Brca1*$^{F/F}$*;Trp53*$^{+/-}$ mice were on average 14-fold larger (5.8% vs. 0.4% spleen/body weight) and 12-fold larger (4.9% vs. 0.4% spleen/body weight) than control mice. Livers were on average 2- (9.0% vs. 4.3% liver/body weight) and 1.9-fold (9.3% vs. 5.0% liver/body weight) larger. <u>Numbers of mice</u>: Controls (n=6), *Mx1-Cre;Brca1*$^{F/insC}$; *Trp53*$^{+/-}$ (n=9), and *Mx1-Cre;Brca1*$^{F/F}$; *Trp53*$^{+/-}$ (n=13). **(E-J)** Representative H&E stained sections of effaced spleens (F,G) and infiltrated liver (I,J) of *Mx1-Cre;Brca1*$^{F/insC}$; *Trp53*$^{+/-}$ and *Mx1-Cre;Brca1*$^{F/F}$; *Trp53*$^{+/-}$ mice compared to control (E,H). **(K)** Flow cytometric analysis of spleen B cells, T cells, and granulocyte/monocyte late progenitors ($Gr^{+}Mac1^{+}$) in control (n=5), *Mx1-Cre;Brca1*$^{F/insC}$; *Trp53*$^{+/-}$ (n=2) and *Mx1-Cre;Brca1*$^{F/F}$; *Trp53*$^{+/-}$ (n=5) mice. Values represent mean ± SD. Statistical significance was assessed using a log rank test or one-way ANOVA followed by Bonferroni correction (*$p < 0.0167$, **$p < 0.003$, ***$p < 0.0003$,****$p < 0.00003$). All mice carry *Mx1-Cre* except controls.

Supplemental Figure 5

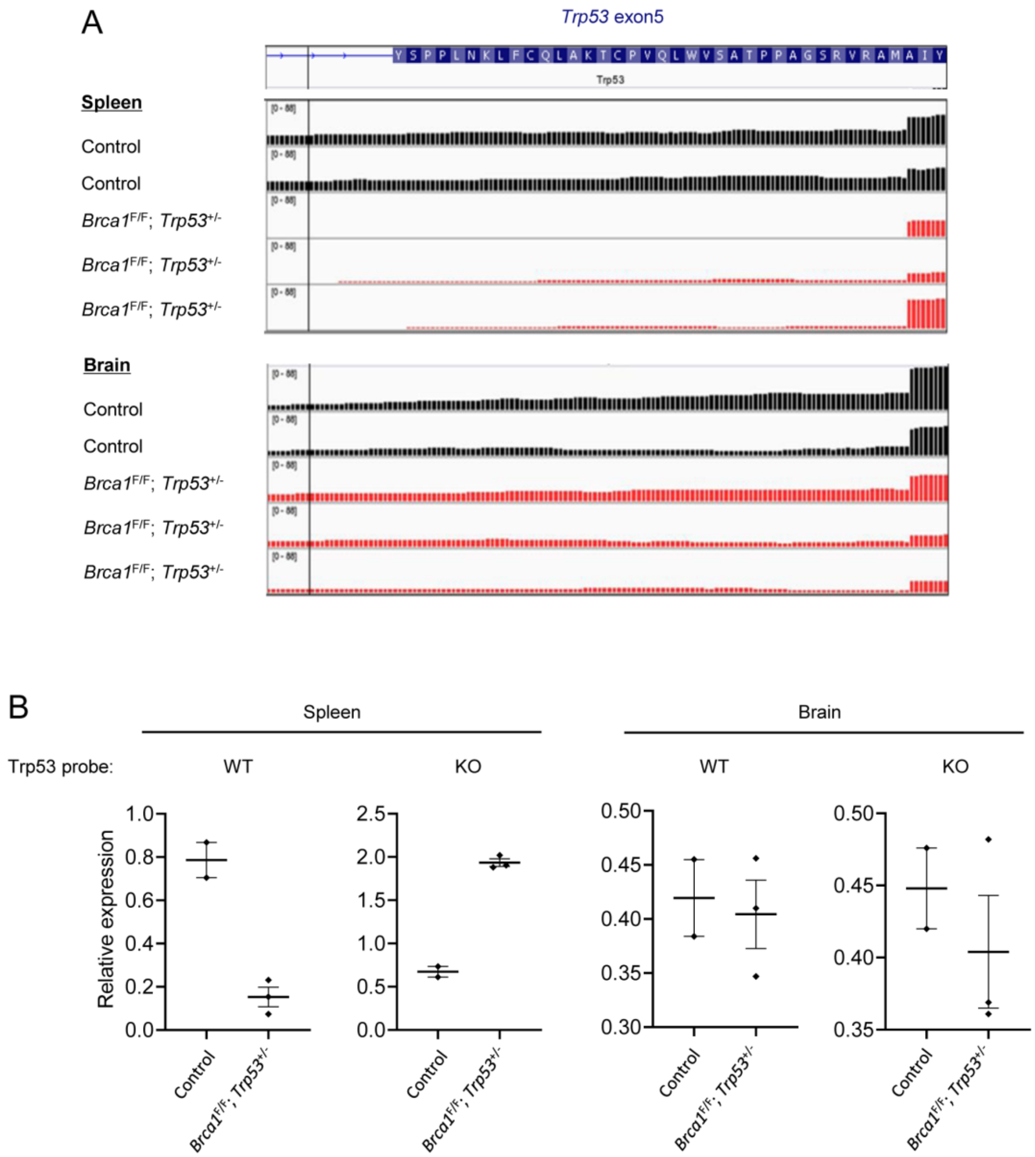

Supplemental Figure 5. Enlarged spleens of diseased *Mx1-Cre; Brca1*$^{F/F}$;*Trp53*$^{+/-}$ mice show *Trp53* LOH. (A) Whole exome analysis show specific loss of heterozygosity in the spleens of diseased *Mx1-Cre;Brca1*$^{F/F}$;*Trp53*$^{+/-}$ mice compared to brains and tissue of control *Brca1*$^{F/F}$;*Trp53*$^{+/-}$ mice. Signal intensity of the genomic region corresponding to the *Trp53* deletion is reduced in *Mx1Cre*;*Brca1*$^{F/F}$;*Trp53*$^{+/-}$ spleens compared to control *Brca1*$^{F/F}$;*Trp53*$^{+/-}$ spleens. No difference in signal between control

Brca1$^{F/F}$;*Trp53*$^{+/-}$ and *Mx1-Cre;Brca1*$^{F/F}$;*Trp53*$^{+/-}$ brains. **(B)** RT-PCR genotyping analysis show decreased wildtype (WT) probe and increased knockout (KO) probe in enlarged *Mx1-Cre;Brca1*$^{F/F}$;*Trp53*$^{+/-}$ spleens compared to control *Brca1*$^{F/F}$;*Trp53*$^{+/-}$ spleens. No difference in brain tissue. Control *Brca1*$^{F/F}$;*Trp53*$^{+/-}$ (n=2) and *Mx1-Cre;Brca1*$^{F/F}$;*Trp53*$^{+/-}$ (n=3).

Supplemental Figure 6

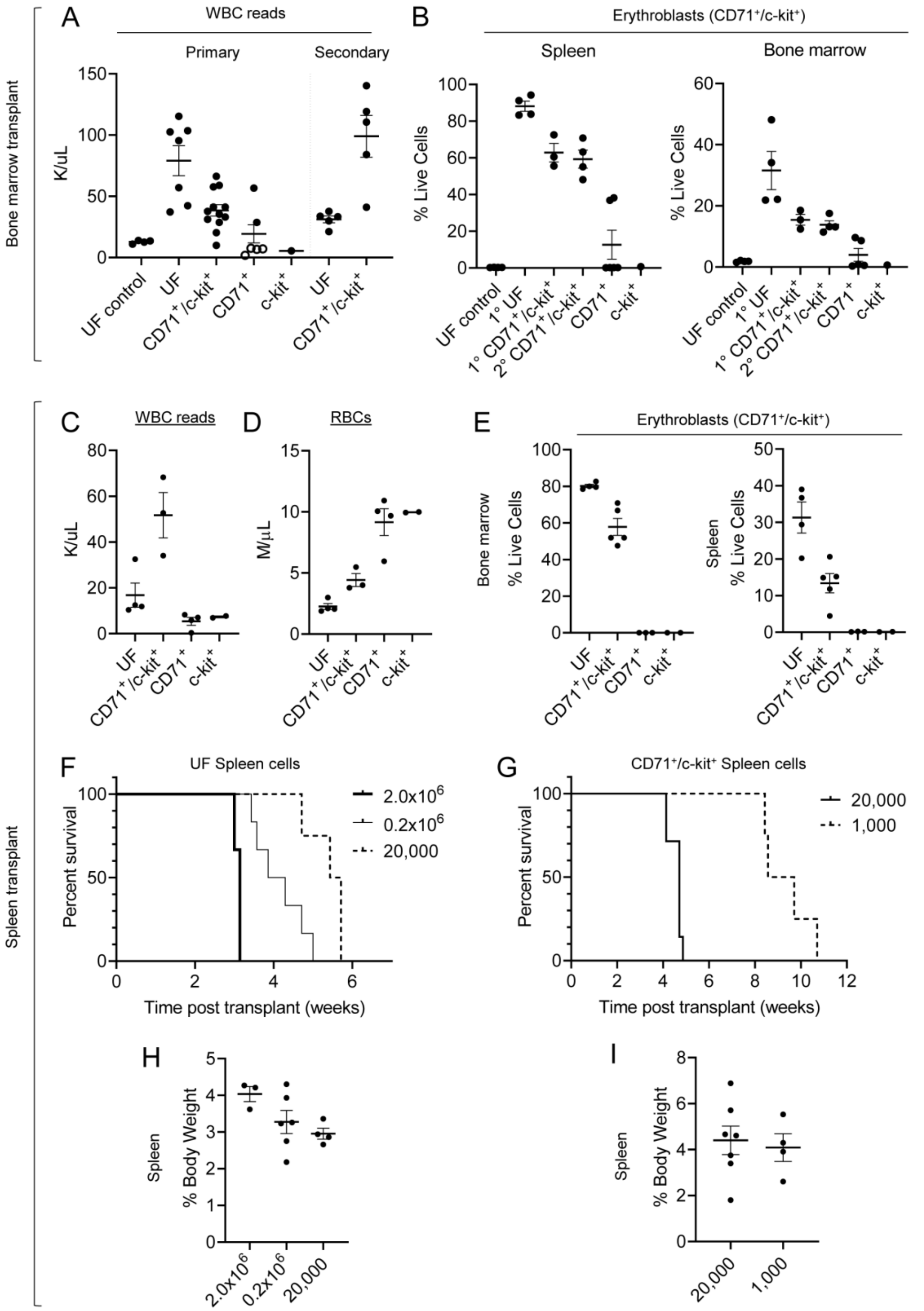

Supplemental Figure 6. Erythroid neoplasia of *Brca1* and *Trp53* double deficiency is transplantable through diseased bone marrow and spleen. (A) Terminal white blood cell (WBC) reads of primary and secondary recipients of control or *Mx1-Cre;Brca1*$^{F/F}$;*Trp53*$^{+/-}$ bone marrow (BM) cells. WBC reads* of CD71+ recipient mice with prolonged survival are marked by open circles. Control UF (n=4) and *Mx1-Cre;Brca1*$^{F/F}$;*Trp53*$^{+/-}$ primary UF (n=7), primary CD71$^+$/c-kit$^+$ (n=12), primary CD71$^+$ (n=7), primary c-kit$^+$ (n=1), secondary UF (n=5), secondary CD71$^+$c-kit$^+$ (n=5). **(B)** Flow cytometric analysis for CD71$^+$/c-kit$^+$ erythroblasts in recipients of control UF (n=4) or *Mx1-Cre;Brca1*$^{F/F}$;*Trp53*$^{+/-}$ bone marrow cells – primary (1°) UF (n=4), 1° CD71$^+$/c-kit$^+$ (n=3), secondary (2°) CD71$^+$/c-kit$^+$ (n=4), 1° CD71$^+$(n=6), 1° c-kit$^+$(n=1). **(C-D)** Terminal white blood cell reads* (WBCs) (C) and red blood cell counts (RBCs) of recipients of *Mx1-Cre;Brca1*$^{F/F}$;*Trp53*$^{+/-}$ spleen cells - UF (n=4-5), CD71$^+$/c-kit$^+$ (n=3), CD71$^+$ (n=4-5), and c-kit$^+$ (n=2). **(E)** Flow cytometric analysis for CD71$^+$/c-kit$^+$ erythroblasts in spleen and bone marrow of mice that received spleen cells. UF (n=4), CD71$^+$/c-kit$^+$ (n=5), CD71$^+$ (n=3), and c-kit$^+$ (n=2). **(F)** Kaplan-Meier curves of overall survival for recipients of 2.0x10^6 (bold solid line, n=3), 0.2x10^6 (solid line, n=6), or 0.02x10^6 (dotted line, n=4) of unfractionated (UF) *Mx1-Cre;Brca1*$^{F/F}$;*Trp53*$^{+/-}$ spleen (SP) cells. Average time to disease is 3.14 weeks, 4.07 weeks, and 5.57 weeks respectively. **(G)** Kaplan-Meier survival curves for recipients of 20,000 (solid line, n=7) or 1,000 (dotted line, n=4) CD71$^+$/c-kit$^+$ spleen cells from *Mx1-Cre;Brca1*$^{F/F}$;*Trp53*$^{+/-}$ mice. **(H)** Elevated terminal spleen weights of recipient mice in (F). **(I)** Elevated terminal spleen weights of recipient mice in (G). * Indicate erythroid blast cells. Values represent mean ± SEM.

Supplemental Figure 7

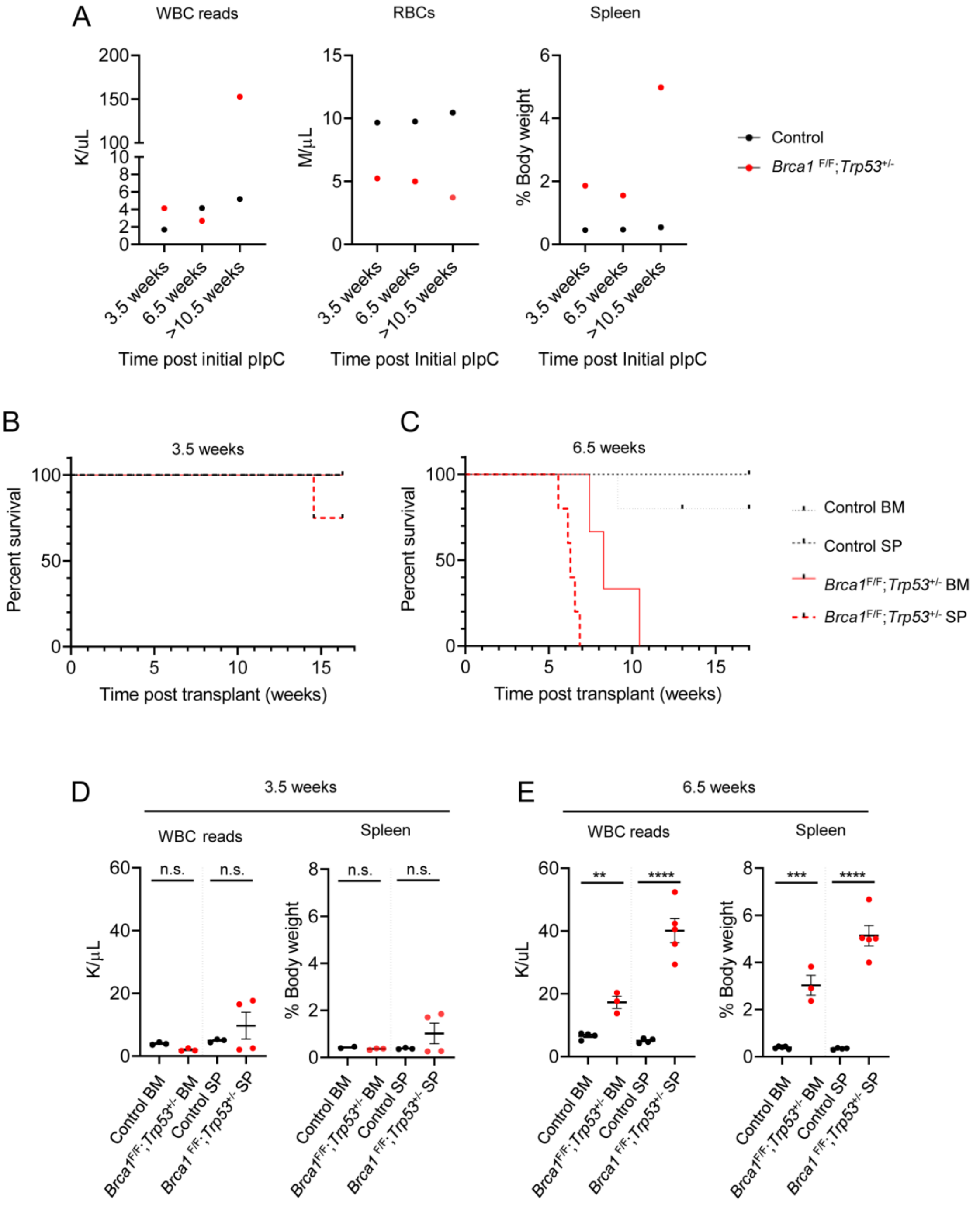

Supplemental Figure 7. ***Brca1/Trp53*** **deficiency-associated erythroleukemia can be transplanted from bone marrow or spleen prior to disease manifestation in peripheral blood (A)** White blood cell reads* (WBCs), red blood cell counts (RBCs) and spleen weights of 3.5-, 6.5-m, and >10.5-week old *Mx1-Cre;Brca1*$^{F/F}$;*Trp53*$^{+/-}$ or control donors. **(B-C)** Kaplan-Meier survival curves of the recipients of 3.5- or 6.5-week bone marrow (BM) or spleen (SP) cells. Control BM (n=5), SP (n=4) and *Mx1-Cre;Brca1*$^{F/F}$;*Trp53*$^{+/-}$ BM (n=3), SP (n=5). **(D-E)** Terminal white blood cell reads* (WBCs) and spleen weights of recipient mice. * Indicate erythroid blast cells. Values represent mean ± SEM. Statistical significance was assessed using a two-tailed Student's t test (*p < 0.05, **p < 0.01, ***p < 0.001,****p < 0.0001). All mice carry *Mx1-Cre* except controls.

Supplemental Figure 8

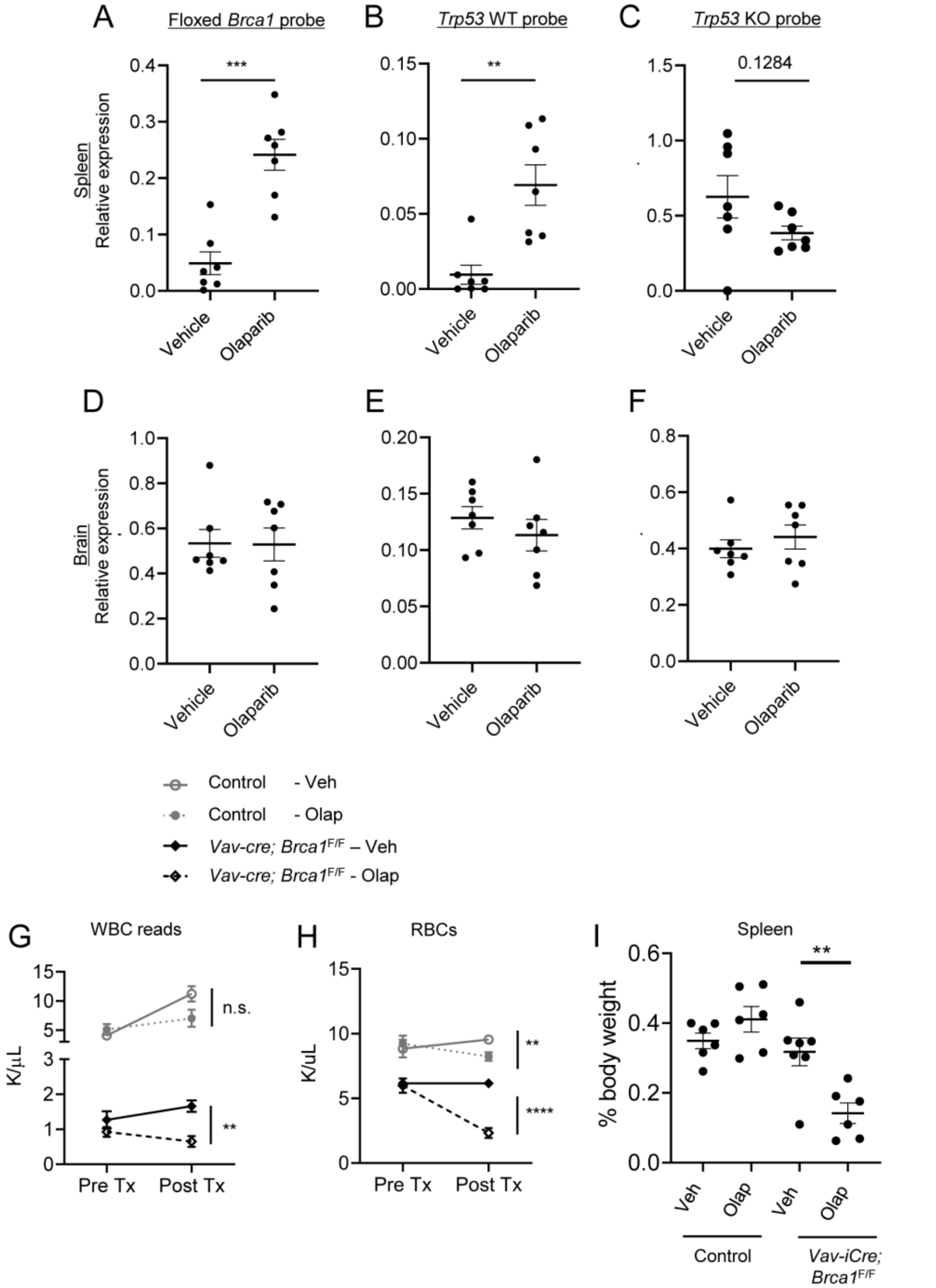

Supplemental Figure 8. *Brca1* deficient hematopoietic cells are sensitive to PARP inhibitor olaparib. (A-F) The signal levels of the probes that recognize unrecombined floxed *Brca1* (A) and wild-type *Trp53* (B) are significantly higher and the probe that recognize *Trp53* knockout (C) lower in olaparib-treated spleens (n=7) compared to vehicle-treated spleens (n=7). No differences seen between the two treatment groups in brain tissue (D-F). **(G-H)** Peripheral blood counts before (Pre Tx) and after (Post Tx) 10 daily treatments of olaparib (50mg/kg) or vehicle. Reduced white blood cell (WBC) reads* (2.55-fold) and red blood cell (RBC) (2.64-fold) counts in olaparib-treated *Vavi-Cre;Brca1*$^{F/F}$ mice (n=5) compared to vehicle-treated *Vavi-Cre;Brca1*$^{F/F}$ mice (n=7). No significant decrease of WBC reads* and modest decrease of RBC counts seen in olaparib-treated control mice (n=6) compared to vehicle-treated control mice (n=6). * Indicate erythroid blast cells. **(I)** Reduced spleen weights of olaparib-treated *Vavi-Cre;Brca1*$^{F/F}$ mice (n=6) compared to vehicle-treated *Vavi-Cre;Brca1*$^{F/F}$ mice (n=7). No difference in spleen weights between olaparib- and vehicle-treated control mice (n=6). Values represent mean ± SEM. Statistical significance was assessed using a two-tailed Student's t test (*$p < 0.05$, **$p < 0.01$, ***$p < 0.001$,****$p < 0.0001$). All mice carry *Vav1-iCre* except controls.

Supplemental Figure 9

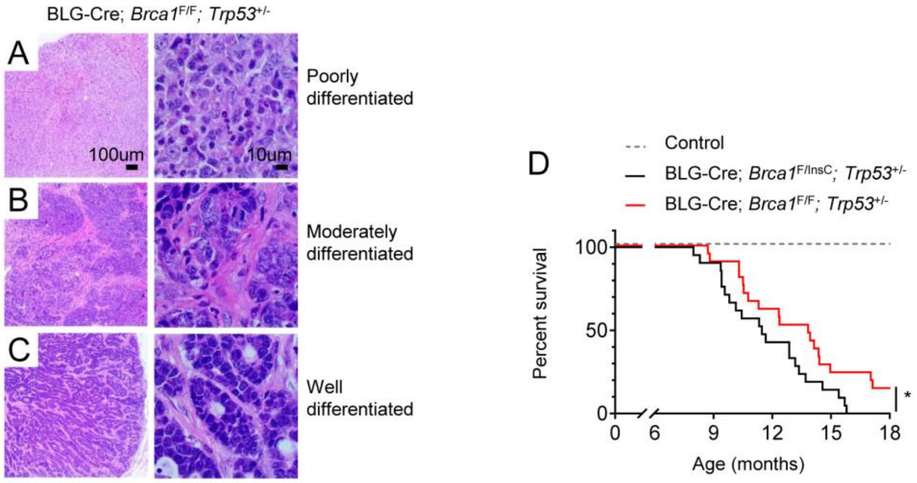

Supplemental Figure 9. *BLG-cre;Brca1*$^{F/F}$;*Trp53*$^{+/-}$ and *BLG-cre;Brca1*$^{F/insC}$;*Trp53*$^{+/-}$ mice develop mammary tumors following a long latency. (A-C) Histopathology of *BLG-cre;Brca1*$^{F/F}$;*Trp53*$^{+/-}$ tumors categorized as poorly differentiated (A), moderately differentiated (B), or well differentiated (C). **(D)** Kaplan-Meier curves for survival of control (dashed black, n=15), *BLG-cre;Brca1*$^{F/F}$;*Trp53*$^{+/-}$ (solid black, n=20), and *BLG-cre;Brca1*$^{F/insC}$;*Trp53*$^{+/-}$ (solid red, n=21) mice. Average time to maximum tumor *BLG-cre*;*Brca1*$^{F/F}$;*Trp53*$^{+/-}$ 11.5 months vs. *BLG-cre*;*Brca1*$^{F/insC}$;*Trp53*$^{+/-}$ 13.9 months, p=0.032). Statistical significance was assessed using a log-rank test. Controls were paired littermates of various genotypes, all without a *BLG-Cre* allele.

Supplemental Table 1. Antibodies used in flow cytometric analysis.

Antibody	Clone	Conjugate	Catalog no.	Provider
Sca1	D7	PE-Cy7	108113	BioLegend
CD117 (c-kit)	2B8	APC-Cy7	105825	BioLegend
CD117 (c-kit)	2B8	APC-eFluor780	47-1171-82	Invitrogen
CD117 (c-kit)	2B8	PE-Cy7	105813	BioLegend
CD48	HM48-1	APC	103411	BioLegend
CD150	TC15-12F12.2	PE	115903	BioLegend
CD16/32	93	Alexa Fluor 700	56-0161-82	Invitrogen
CD34	RAM34	FITC	11-0341-82	Invitrogen
CD3	17A2	Alexa Fluor 700	56-0032-82	Invitrogen
CD3	17A2	PE	100205	BioLegend
CD3ε	142-2C11	Biotin	100301	BioLegend
CD4	GK1.5	PE	12-0041-82	eBioscience
CD4	GK1.5	FITC	100405	BioLegend
CD4	GK1.5	Biotin	100403	BioLegend
CD5	53-7.3	Biotin	100603	BioLegend
CD8a	53-6.7	Biotin	100703	BioLegend
CD8a	53-6.7	FITC	100705	BioLegend
Ter119	TER-119	APC	116211	BioLegend
Ter119	TER-119	Biotin	116203	BioLegend
CD45	30-F11	Alexa Fluor 700	103127	BioLegend
CD45R (B220)	RA3-6B2	PE	103207	BioLegend
CD45R (B220)	RA3-6B2	PerCP-Cy5.5	65-0452	TONBO
CD45R (B220)	RA3-6B2	Biotin	103203	BioLegend
Gr-1 (Ly-6G)	RB6-8C5	PE	108407	BioLegend
Gr-1 (Ly-6G)	RB6-8C5	PE-Cy7	108415	BioLegend
Gr-1 (Ly-6G)	RB6-8C5	Biotin	108403	BioLegend
CD11b (Mac-1)	M1/70	APC-eFluor780	47-0112-82	Invitrogen
CD11b (Mac-1)	M1/70	PE	553311	BD Pharmigen
CD11b (Mac-1)	M1/70	Biotin	101230	BioLegend
CD71	RI7217	BV421	113813	BioLegend
Streptavidin		PE-CF594	562318	BD Biosciences
